# Mind the road: attention related neuromarkers during automated and manual simulated driving captured with a new mobile EEG sensor system

**DOI:** 10.3389/fnrgo.2025.1542379

**Published:** 2025-03-12

**Authors:** Joanna Elizabeth Mary Scanlon, Daniel Küppers, Anneke Büürma, Axel Heinrich Winneke

**Affiliations:** ^1^Fraunhofer Institute for Digital Media Technology, Branch Hearing, Speech and Audio Technology, Oldenburg, Germany; ^2^Institute of Cognitive Science, University of Osnabrück, Osnabrück, Germany

**Keywords:** driver monitoring system (DMS), vigilance, mobile EEG measurement system, autonomous driving, advanced driver assistance system (ADAS), mental state

## Abstract

**Background:**

Decline in vigilance due to fatigue is a common concern in traffic safety. Partially automated driving (PAD) systems can aid driving but decrease the driver's vigilance over time, due to reduced task engagement. Mobile EEG solutions can obtain neural information while operating a vehicle. The purpose of this study was to investigate how the behavior and brain activity associated with vigilance (i.e., alpha, beta and theta power) differs between PAD and manual driving, as well as changes over time, and how these effects can be detected using two different EEG systems.

**Methods:**

Twenty-eight participants performed two 1-h simulated driving tasks, while wearing both a standard 24 channel EEG cap and a newly developed, unobtrusive and easy to apply 10 channel mobile EEG sensor-grid system. One scenario required manual control of the vehicle (manual) while the other required only monitoring the vehicle (PAD). Additionally, lane deviation, percentage eye-closure (PERCLOS) and subjective ratings of workload, fatigue and stress were obtained.

**Results:**

Alpha, beta and theta power of the EEG as well as PERCLOS were higher in the PAD condition and increased over time in both conditions. The same spectral EEG effects were evident in both EEG systems. Lane deviation as an index of driving performance in the manual driving condition increased over time.

**Conclusion:**

These effects indicate significant increases in fatigue and vigilance decrement over time while driving, and overall higher levels of fatigue and vigilance decrement associated with PAD. The EEG measures revealed significant effects earlier than the behavioral measures, demonstrating that EEG might allow faster detection of decreased vigilance than behavioral driving measures. This new, mobile EEG-grid system could be used to evaluate and improve driver monitoring systems in the field or even be used in the future as additional sensor to inform drivers of critical changes in their level of vigilance. In addition to driving, further areas of application for this EEG-sensor grid are safety critical work environments where vigilance monitoring is pivotal.

## Introduction

Driver fatigue and the associated decline in vigilance is a common concern when it comes to traffic safety. According to the American Automobile Association (AAA) Foundation for Traffic Safety, driver fatigue contributes to about 100,000 crashes, 71,000 injuries and 1,550 deaths each year in the United States alone (Owens et al., 2018). In Germany, current figures demonstrate similar findings (relative to population) that there were 1,507 fatigue-related accidents causing injury in 2021, with unreported cases expected to be significantly higher (ADAC, 2022). Studies of traffic accident casualties have shown that ~32% of U.S. drivers will drive a vehicle while in a fatigued state at least once per month (Zhang et al., 2016). Additionally, according to a survey by the German Road Safety Council (Deutscher Verkehrssicherheitsrat, 2016), 26% of surveyed drivers admitted to falling asleep at the wheel at least once. As if the consequences for the individuals involved in drowsiness related accidents are not bad enough, this has led to a cost of more than $100 billion annually, not including property damage (Higgins et al., 2017). At the same time, vehicles equipped with automated driving systems are becoming more popular (e.g., Department U. S. of Transportation, 2018). While these systems themselves do not experience fatigue in the way that humans do, it is unclear whether these systems can solve the problem of driver fatigue.

Currently, the standard for automated vehicle technology is that vehicles are “partially automated” [Society of Automotive Engineers (SAE) Level 2]. This means that the driver is not responsible for the car's longitudinal or lateral position, but instead must supervise the automated driving system (SAE International, 2018). This is because, while automated vehicles are steadily improving, they still suffer from imperfections, which may lead to failures in detecting and responding to dangerous hazards on the roadway such as pedestrians (National Transportation Safety Board, 2019a), crossing traffic (National Highway Traffic Safety Administration, 2017), or stopped vehicles (National Transportation Safety Board, 2019b). Therefore, the driver must remain vigilant and attentive to the road, in order to act as a failsafe in emergencies, and take over control in the case that the vehicle does not respond correctly (Greenlee et al., 2022; National Highway Traffic Safety Administration, 2017). Therefore, we refer to this type of driving as partially automated driving (PAD).

Although PAD reduces the driver's workload by changing their role to only a failsafe instead of the main controller of the vehicle, there is some evidence to show that PAD can have a negative impact on the driver's vigilance. Vigilance is defined as the ability to maintain the necessary level of sustained attention while performing a task in order to respond correctly when the situation requires a response (Davies and Parasuraman, 1982; Parasuraman, 1986; Warm and Parasuraman, 2008). Vigilance to sustained tasks such as PAD tends to decrease over time (Mackworth, 1948), due to cognitive underload, in which one experiences a low amount of cognitive demand, likely due to lower levels of engagement with the driving environment (McWilliams and Ward, 2021). Cognitive underload is especially common when performing monotonous, low demand driving scenarios (Körber et al., 2015a; McWilliams and Ward, 2021). In previous studies, Greenlee et al. (2018, 2019, 2022) demonstrated that the likelihood of detecting hazards decreases over time for drivers in automated simulated vehicles. Therefore, in partially automated vehicles, the driver's ability to monitor the vehicle and the traffic to prevent collisions will decrease as a function of time (Greenlee et al., 2018, 2019, 2022; Körber et al., 2015a; Mok et al., 2015). Cognitive underload can then lead to passive fatigue, which is the depletion of attentional resources due to low task demands over time (Desmond and Hancock, 2000; McWilliams and Ward, 2021; Saxby et al., 2013). Previous studies have indicated that monitoring a PAD simulation can induce passive fatigue (Saxby et al., 2013), and stress (Funke et al., 2007), and is cognitively demanding enough to lead to reduced vigilance over time (Greenlee et al., 2018, 2019). In other words, the state of fatigue caused by a monotonous task with low cognitive demand such as driving leads to reduced vigilance, and therefore for this study it is necessary to acknowledge both of these concepts in the context of driving.

Increasing time devoted to a driving task tends to decrease driving performance in measures such as the standard deviation of lateral lane position (i.e., how much the vehicle weaves) and therefore driving vigilance (van der Hulst et al., 2001; Verster and Roth, 2013; Ting et al., 2008; Thiffault and Bergeron, 2003; Philip et al., 2005). This vigilance decrement may be enhanced in PAD because the reduced driving workload reduces driver engagement (McWilliams and Ward, 2021). In one study, when the same 40-min vigilance task was compared between partially automated and manual simulated driving, the PAD condition showed more severe vigilance-related decrements (Greenlee et al., 2022). While participants in both conditions showed a decreasing percentage of correct target detections over time, only the automated condition showed decreased sensitivity to the hazardous stimuli and increasing tendency for false alarms. In the manual condition, these measures remained consistent over time (Greenlee et al., 2022).

Other studies comparing PAD to manual driving have observed PAD conditions to show slower response times to a concurrent task (Biondi et al., 2018; Neubauer et al., 2012), as well as increased heart rate variability (Biondi et al., 2018), increased self-reported distress and decreased engagement (Neubauer et al., 2012). Altogether, these studies indicate that PAD does not necessarily make driving safer, as reduced vigilance can possibly lead to collisions in real world driving situations when the driver has to take over control of the vehicle.

Since lapses in attention and vigilance during driving can be dangerous, some studies have suggested the use of artificial intelligence systems to detect such lapses in attention (Simon et al., 2011; Arefnezhad et al., 2019; Awais et al., 2014; Arefnezhad et al., 2022; Stancin et al., 2021; Zhou et al., 2021). Systems that analyze driving behavior have been suggested (Arefnezhad et al., 2019), since this is arguably one of the simplest ways to measure driving vigilance (Verster and Roth, 2013), when the driver is actively controlling the car. However, driver behavior can be affected heavily by external factors, such as obstacles and features of the roadway, and also driving performance information would be unavailable in automated conditions. Additionally, with the human brain as source for cognitive functioning, measuring brain activity may ideally allow attentional changes to be detected earlier than with changes in behavior or observable indices such as drooping eye lids or increased blinking rate. For example, parietal and occipital EEG alpha activity (8–12 Hz) as well as frontal channel theta power (4–8 Hz) have been shown to increase with mental fatigue (Wascher et al., 2014; Dehais et al., 2020; for a review see Borghini et al., 2014 and Tran et al., 2020) with theta activity and changes thereof reflecting workload in a more complex multitasking environment (Sciaraffa et al., 2022). Additionally, frontal beta power increases with time-on-task as increasing levels of mental effort are required to maintain vigilance (Pershin et al., 2023). Therefore, changes in spectral EEG activity such as in alpha, beta and theta power have been identified as neurophysiological biomarkers of vigilance and drowsiness or fatigue during driving (Awais et al., 2014; Arefnezhad et al., 2022; Sciaraffa et al., 2022). Several studies have used these neuro-signals to attempt monitoring a driver's spectral brain activity during both manual driving (Awais et al., 2014; Arefnezhad et al., 2022; Simon et al., 2011; for a review, see Stancin et al., 2021) and PAD (Zhou et al., 2021). Particularly frequency-based measures are of interest for real-world settings as they do not rely on frequently repeating external events which is the case for ERP-based approaches.

Another scientifically established method that has been used to observe drowsiness and vigilance decrements is to assess PERCLOS (PERcentage of eye CLOSure) through eye tracking (Abe et al., 2011; Arefnezhad et al., 2022; McWilliams and Ward, 2021). PERCLOS has also been shown to increase with time on task during PAD (Heikoop et al., 2017) and has been used as a “ground truth” for assessing driver drowsiness in some cases (Arefnezhad et al., 2022). Similarly, eye blinks have been suggested as another way to detect drowsiness during driving as blink rate increases with time on task and when individuals are drowsy (Cori et al., 2021). Additionally, increase in heart rate variability (Heikoop et al., 2017), as well as decreases in heart rate (Pattyn et al., 2008; Heikoop et al., 2017) have also been shown with increasing time on task during vigilance tasks. While these previous studies show that various physiological measures can demonstrate decreases in vigilance during driving, there are currently no studies, of which we are aware, that have observed these physiological measures in a comparison between partially automated and manual driving in a long monotonous simulated driving scenario.

Furthermore, as indicated above EEG promises to be a particularly sensitive method to detect changes in vigilance as it measures the source of cognitive function, namely the brain itself (for reviews, see Peng et al., 2022; Halin et al., 2021 and Stancin et al., 2021). Recent reviews suggest that EEG offers the most important source of data for successful detection of driver drowsiness (Stancin et al., 2021) and is a gold standard for monitoring perception during driving which is influenced by attentional processes and fatigue (Peng et al., 2022). That being said, one of the challenges associated with this technology is its limited mobility and cumbersome setup. To overcome these limitations, recent developments in sensor technology could provide a solution to bridge the gap between lab-bound scientific experiments and real-world application. Based on the so-called cEEGrids (Bleichner and Debener, 2017; Debener et al., 2015) the trEEGrid was developed to go beyond around-the-ear EEG to provide long-term, high-quality EEG, EOG and EMG signals at low impedances (Da Silva Souto et al., 2021, 2022). Due to their soft, flexible material, the trEEGrids offer very high user comfort with minimal setup time. Originally designed for sleep research, a new trEEGrid variant was designed for the current study to record high-quality EEG and EOG with an unobtrusive, comfortable, and easy-to-apply EEG sensor system to be worn around the ear. Unlike the original trEEGrid, the new variant does not have electrodes along the jaw. This is the first time this new variant of the trEEGrid was used for a mainly cognitive task outside the area of sleep research.

In the current study, our goal was to investigate the best features which could be used to detect changes in vigilance in a simulated driving scenario. We aimed to observe the effects of driving both partially-autonomously and manually, over time, while observing various measures including PERCLOS, brain activity, heart rate variability as well as some behavioral and subjective measures. Participants drove an hour-long driving scenario in a driving simulator in both autonomous and manual mode (i.e., 1 h each), while being recorded by two types of EEG apparatuses (standard EEG cap and custom EEG grid) as well as using an eye tracking, and electrocardiogram (ECG) instruments. Between driving conditions, participants performed a psychomotor vigilance task (PVT) and filled out questionnaires to rate their subjective levels of alertness/sleepiness, task-load, engagement, and stress. The new design version of the trEEGrid system was used as a small and highly mobile brain measurement, ideal for real world application, while the EEG cap served as a control, or reference system. Our first hypothesis was that the PAD condition will show lower levels of self-reported alertness as well as physiological fatigue and vigilance decrement (e.g., higher PERCLOS, and higher levels of alpha and beta power), due to the decreased level of engagement required for this task. Our second hypothesis was that in both scenarios, measures of vigilance decrement and fatigue will increase over time, as increasing time on task tends to lead to increased fatigue. Our third hypothesis was that vigilance and fatigue related changes are detected earlier in EEG based signals as opposed to other parameters such as driving performance or PERCLOS. The fourth hypothesis was that the EEG patterns and statistical effects would be the same in both EEG sensor systems.

## Materials and methods

### Participants

Data were collected from 28 participants [age: 20–60 years, mean: 26.6, standard deviation (SD): 7.3] out of which 10 (= 35.71%) were female and 2 (= 7.14%) were left-handed. All of them were recruited from the online forum of the Carl von Ossietzky University of Oldenburg.

All participants reported normal hearing and normal or corrected-to normal vision. All of them possessed a valid driver's license (years of possession: 1–42 years, mean: 7.9, SD: 7.8). None of the participants claimed to have medical implants or magnetizable metal implanted in the body/skull. In addition, none of the subjects reported to be pregnant or to have neurological disorders or seizure disorders. Also, no participant took medications that limit driving ability.

The participants gave their informed consent before the start of the study and received 12 € per hour for their participation in the experiment. The experiment was approved by the ethics committee of the Carl von Ossietzky University in Oldenburg and the dataset was collected in accordance with established research protocols and ethical guidelines.

### EEG devices

**Figure 1:**
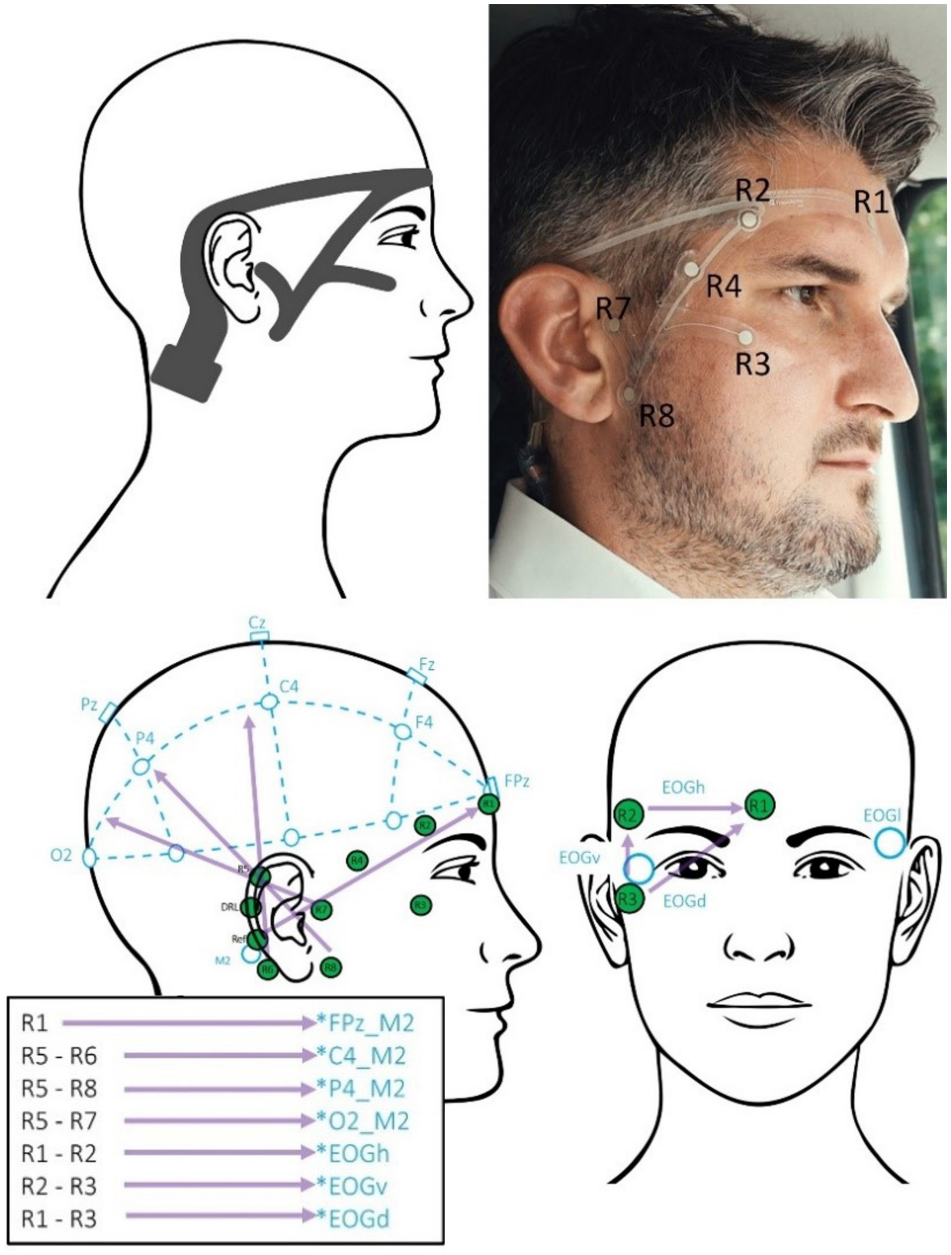
**(Top)**: Left: drawing of the trEEGrid grid designed for this study including connector endpiece. Right: trEEGrid grid when attached to a user. © Leona Hoffmann, Fraunhofer IDMT. **(Bottom)**: Left: lateral view of the right side of the face and right frontal view of the face. Electrode positions and corresponding labels of the standard 10–20 system in blue with reference to M2 on the right mastoid behind the ear. Electrode positions of the trEEGgrid in green [DRL = ground (Driven Right Leg); Ref = reference]. The purple-colored vectors illustrate the channel combinations to approximate 10–20 electrode locations. The box contains supplementary information how channels can be combined to approximate 10–20 electrodes referenced to the mastoid reference electrode.

EEG was recorded with two EEG systems simultaneously. Therefore, participants were first fitted with an EEG-trEEGrid with 10 channels (layout shown in Figure 1). Then, an EEG cap with 24 channels arranged according to the 10–20 system was placed over the EEG-trEEGrid. The electrodes of both EEG systems were prepared with abrasive electrolyte gel (Abralyt HiCl, Easycap, Germany).

### trEEGrid

By applying a method of spatial approximation, it has been shown that different channel combinations on the EEG-grid allow for approximating the EEG signal from typical 10–20 channels located in hairy areas on the scalp referenced to the right trEEGrid mastoid channel M2 (Bleichner et al., 2016). This approach thereby yields the following “virtual” grid channels [similar to Da Silva Souto et al., 2021; see Figure 1 (bottom)]: FPz_M2, C4_M2, P4_M2, O2_M2, as well as horizontal, vertical and diagonal EOGs. To avoid confusion with actual 10–20 cap channels, trEEGrid channels will be labeled with the affix “_M2” to indicate the virtual approximations referenced to the trEEGrid right mastoid “M2.” Previously it was shown that EEG parameters recorded with the trEEGrid and data recorded from standard 10–20 locations (Da Silva et al., 2022) correlate significantly, supporting the use of this new technology for unobtrusive EEG recordings.

### EEG cap

The cap EEG system consisted of 24 Ag/AgCl ring electrodes (EasyCap GmbH, Brain Products GmbH) according to the 10–20 system (channels Fp1, Fp2, Fz, F7, F8, FC1, FC2, Cz, C3, C4, T7, T8, CPz, CP1, CP2, CP5, CP6, Pz, P3, P4, O1, O2, M1, M2). In case of overlapping electrodes between cap and grid, the cap electrode was loosened and attached slightly next to its actual position using a suitable ring-shaped adhesive tape. This was the case for less than five participants involving at least one electrode of Fp2, T8 or M2 and did not influence the signals. Both systems (cap and grid) were each connected to separate wireless Smarting “mobiSleep” amplifiers (mBrainTrain, Serbia), transmitting the data via Bluetooth at a sampling rate of 250 Hz to a PC (Dell Precision 3660 Tower; trEEGrid) or laptop (Dell Latitude 5280; EEG-Cap) using the Smarting Streamer application for Windows (mBrainTrain, Serbia). Impedance values for both the cap and the grid were kept below 15 kOhm and the EEG-signal quality was checked by visual inspection of the EEG data.

### Additional measurement equipment

In addition to the EEG systems described above, further devices were used to acquire data from the participants. A Tobii Pro Spectrum 600 stationary eyetracker (Tobii, Stockholm, Sweden) was used to record gaze coordinates and eye openness information at a 600 Hz sampling rate. To measure heart rate variability, a Zephyr Bioharness 3.0 chest belt (Zephyr Technology, Boulder, USA) was used which transmitted data at a sampling rate of 250 Hz. A photodiode was connected to a LabStreamerBox (Neurobehavioral Systems, USA) to provide exact stimulus timing data at 12 kHz.

### Driving simulator set-up

**Figure 2:**
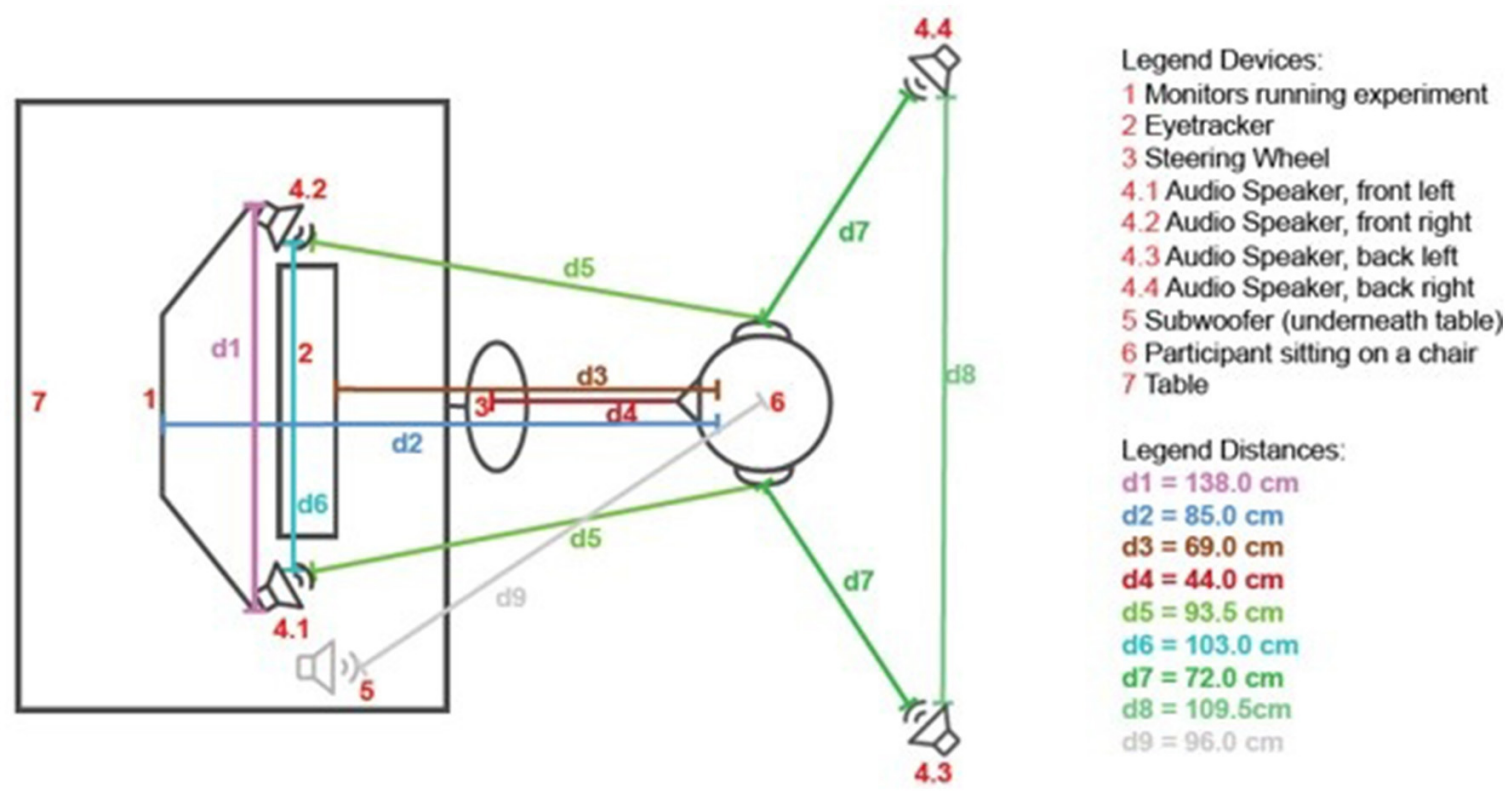
Schematic visualization of the device setup in the room. Additional measures: table height = 72.0 cm, height of speakers (4.3/4.4) = 102.0 cm, distance eyetracker to tabletop = 24.0 cm, angle eyetracker = 25.5°.

The driving simulator was set up in a room at the Fraunhofer IDMT in Oldenburg. Carnetsoft driving simulator software (Carnetsoft BV, Groningen, Netherlands) was used for the driving simulation and driving task presentation. In Figure 2, a schematic representation of the experimental setup is depicted. The participants occupied a fixed position in front of a table, where they were positioned to observe three interconnected monitors [Dell Ultra Sharp U2422H 24 inches (1920 × 1080); denoted as 1]. These screens were connected to a computer [Dell Precision 3,660 Tower (Intel Core i9)] and displayed the simulated driving environment, imitating a 210 degrees horizontal field of view (including three rear view mirrors).

Directly in front of the screens, the stationary eyetracker Tobii Pro Spectrum 600 (2) was placed. The front of the eyetracker maintained a distance of 69.0 cm (d3) from the participant's eyes and was inclined at an angle of 25.5°. The Logitech G29 steering wheel (Logitech, Switzerland; (3) was fixed to the table in front of the participant.

The auditory experience was realized with four loudspeakers and a subwoofer surrounding the participant (Teufel Concept E 450 Digital 5.1). Two speakers were positioned on the left and right sides of the eyetracker, directly beneath the screens and two audio speakers were situated behind the participant, positioned at a height of 102 cm and oriented toward the participant's ears. Complementing the audio setup, a subwoofer (5) was situated beneath the table. All devices, except for audio speakers 4.3 and 4.4, were positioned on a table (7) with a standardized height of 72 cm.

### Recording equipment and setup

**Figure 3:**
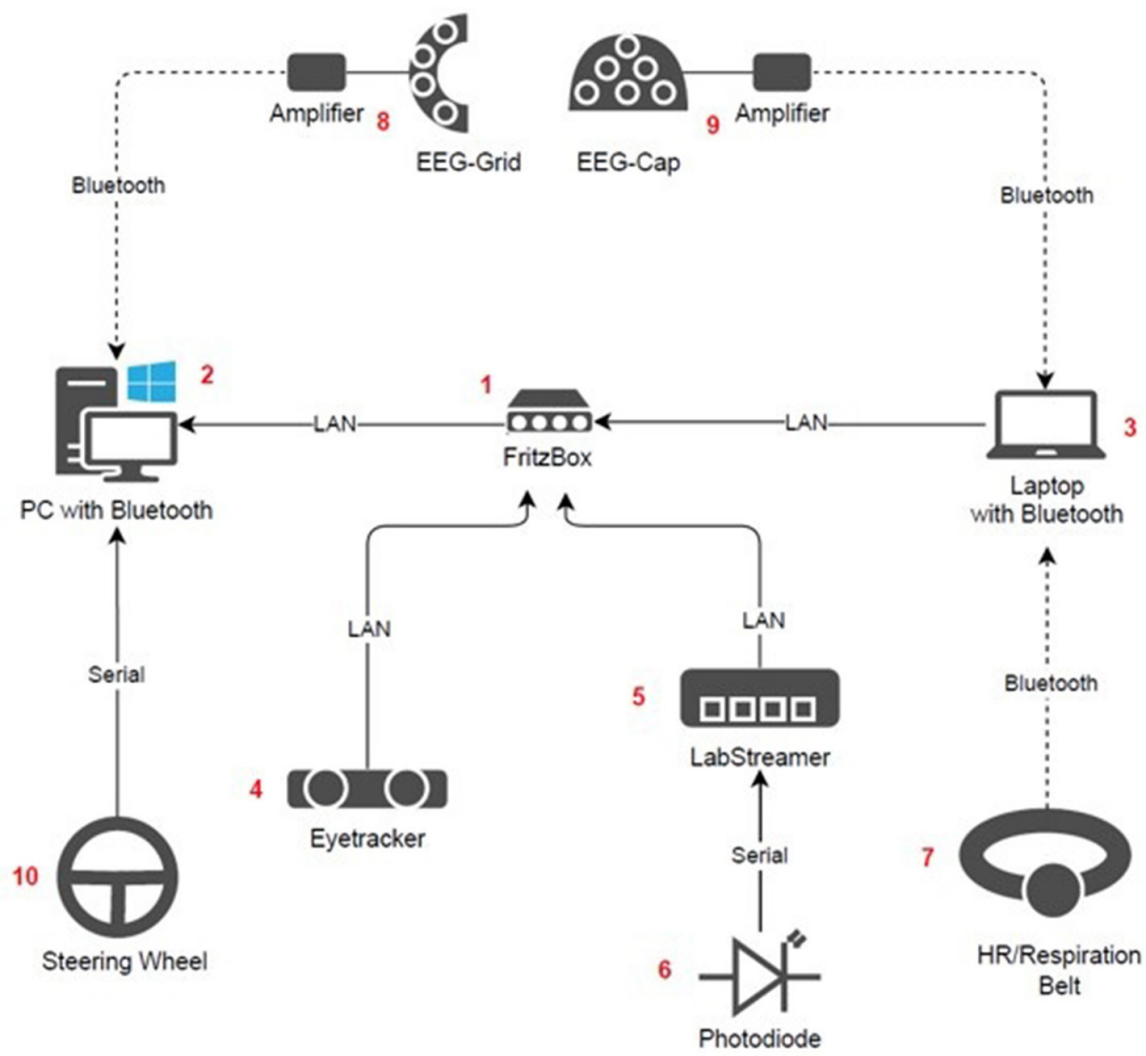
Schematic visualization of the devices and the dataflow connections. The arrow directions describe the dataflow of the collected data.

A schematic visualization of the devices used in the experiment and the corresponding data streams are shown in Figure 3. All data streams were temporally synchronized and stored via LabStreamingLayer protocol (LSL; Kothe et al., 2014). The stationary eyetracker (4) transmitted gaze coordinates and eye openness information via two LSL streams. To monitor and extract the exact timing of the cars appearing in the virtual environment a photodiode was placed on the left side of the central monitor. When a car appeared on the screen, a white dot appeared simultaneously directly under the photodiode. The photodiode (6) and white spot on the screen were both covered with black electrical tape, blending into the black sky of the driving scenario. The photodiode was connected to a LabStreamerBox (Neurobehavioral Systems, USA, 5) to provide stimulus timing data via an LSL stream. The chest belt sent ECG data via Bluetooth to a laptop (3).

A router [Fritz!Box 7530 AX (1; AVM Computersysteme Vertriebs GmbH, Germany)] was installed which received most of the data streams via a Local Area Network (LAN) connection. The router was connected to a laptop (3) via LAN, also receiving data via bluetooth directly from the Zephyr Bioharness 3.0 belt (7) and one EEG stream from the Smarting “Sleep” amplifier connected to the cap (9) EEG system. Data from the eyetracker were also sent to the router via LAN.

From the router, all data streams were sent to a computer (2) via LAN. This computer further recorded one stream from the second Smarting “mobiSleep” amplifier connected to the trEEGrid (8) as well as behavioral data from the driving simulator. The driving simulator data consisted of two streams: one containing information on sporadic events such as stimuli appearing (e.g., cars), responses to stimuli (i.e., button presses on the steering wheel), as well as timing and strength of wind gusts (see section *Tasks and Stimuli* below for more information) and one containing continuous (sampled at 10 Hz) information on driving performance (i.e., lateral position relative to right lane center, steering angle, heading angle, velocity and distance traveled in the scenario map). The steering wheel (10) was connected to the PC via a serial connection.

### Subjective evaluations

In addition to physiological and driving data, subjective evaluations in the form of questionnaires regarding subjective sleepiness and alertness [*Stanford Sleepiness Scale* (SSS; Hoddes et al., 1973)], subjective perception of stress and engagement [*Short Stress State Questionnaire* (SSS-Q; Helton and Näswall, 2015)] and perceived workload [*NASA-Task Load Index* (NASA-TLX; Hart, 1986, 2006)] were administered before, after and between driving blocks. For the SSS and the NASA-TLX available translations into German were used, whereas the SSSQ was translated from English into German by the authors. Questionnaires were in reference to the participants' subjective experience during the driving tasks. Furthermore, a brief 5-min computer-based version of the *Psychomotor Vigilance Test* (PVT; Basner et al., 2011; Loh et al., 2004) was administered before, after the first and after the second driving session to measure sustained attention and whether it changes over the duration of the experiment.

### Procedure

All participants came in for one session which lasted between 3.5 and 4 h. In the beginning of the experiment, participants were given enough time to read information and consent documents, as well as an opportunity to ask questions. After informed consent was given, an EEG cap, a trEEGrid and the chest belt ECG sensor were applied. After a short introduction into the experiment, the participant was seated in the driving simulator. Here the participant performed the first 5-min PVT and was asked to fill out the first SSS-Q and SSS. This was followed by a short practice task for the driving simulator. Subsequently, the eyetracker was calibrated, followed by the first 1-h driving scenario, which was either the partially autonomous (PAD) or the manual scenario. The sequence of experimental scenarios was counterbalanced across participants. After the first driving scenario was completed, participants performed a second PVT, SSS-Q, SSS and the first NASA-TLX. Before the second scenario started, the participants could take a short break. On average the break lasted around 4 min and participants remain seated in the driving simulator. After finishing the second scenario, another PVT, SSS-Q, SSS and NASA-TLX was completed.

### Task and stimuli

#### Driving scenarios

**Figure 4:**
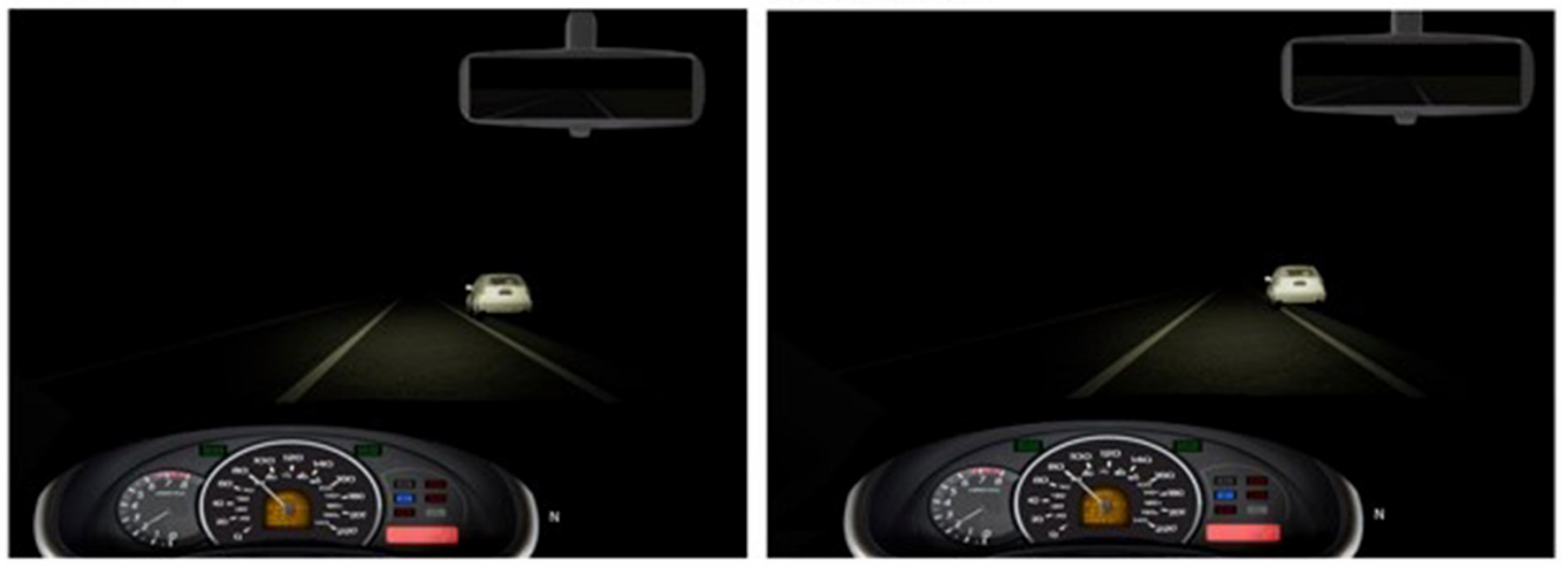
Screenshot (center screen) of the stimuli. **(Left)**: Safely parked car, next to the right lane. **(Right)**: Unsafely parked car protruding into the lane.

The driving simulator task consisted of two nighttime driving sessions each lasting 1 h. The participant could only see about 80–100 m ahead, with no visible scenery to the sides of the roadway (see Figure 4). The simulator task was performed twice, in two conditions: In the manual condition, the participants manually steered the car. Participants drove at a consistent speed of 80 km/h, which was controlled by the simulator to keep the task consistent compared to the autonomous condition (similar to Karthaus et al., 2021). To ensure that the participants were engaged in the driving task, sporadic wind gusts were added to the scenario, requiring participants to actively steer to stay in the center of the lane. The wind gusts were in a direction perpendicular to the driver, at alternating directions every 11.7–14.9 s (260–330 m). Winds blew at a random speed between 0 and 30 km/h, which was enough to move the car over a meter to the left or right and could even cause the car to crash into parked cars in the scenario if no counter-steering was performed. If a crash happened during the manual condition, a “crash” sound was played, and the car was repositioned to the lane center.

In the partially automated (PAD) condition, the same scenario was used, using both automatic speed control and lane keeping. This condition also included wind, for which the automated lane keeping compensated.

#### Hazard detection task

In both conditions, the participants had to monitor the road to respond to a secondary task (i.e., driving was the primary task). For this hazard detection task, white cars were parked along the righthand side of the road either correctly (i.e., the car was fully outside the lines of the road) or in a potentially unsafe position (i.e., the car protruded slightly onto the lane; see screenshots in Figure 3; similar to Greenlee et al., 2018, 2019, 2022). Cars appeared every 4–6 s at about 80 m ahead of the participant and remained visible for 3.5 s, until the vehicle passed them. 12–15% cars were parked unsafely, with no two unsafe cars being parked subsequently. The participants were asked to respond to the unsafely parked cars by pushing a button on the steering wheel with their left hand. The hazard detection task started about 25 seconds (550 m) after the start of the scenario. The hazard detection task was based on a task used by Greenlee et al. (2018, 2019, 2022), with some adjustments for our purposes. This task was originally intended to be an in-scenario oddball task for which we would observe event related potentials (ERPs). However, no significant differences were found in the ERPs between the conditions or over time, so this part of the study was removed.

#### Practice scenario

Before starting the experimental tasks, participants first had to perform a practice driving simulator task. This task lasted ~4 min and consisted of the same car position task as the main task, with the steering set to manual mode. In the practice trial, however, the percentage of unsafe cars was increased to 20%, to ensure sufficient experience detecting the difference between safely and unsafely parked cars. The participant could then ask any questions they had about the task and repeat the practice if necessary.

### Data analysis

#### Data preprocessing

The EEG preprocessing, data analysis and figures were implemented in Python, using various external libraries including MNE (MNE Developers, 2023), NumPy (Van Der Walt et al., 2011), Scipy (Pedregosa et al., 2011), CSV, Math, Pandas (McKinney, 2011) and Matplotlib (Ari and Ustazhanov, 2014). Some figures (scatterplots) were created in Excel. Initially, photodiode data was used to ensure correct timing of the event markers from the driving simulator. Then, cap and trEEGrid data were temporally synchronized, ensuring that the data initiation and termination occurred simultaneously. There was a small amount of data loss (mean PAD: 0.46%, Manual: 0.75%) in a few subjects, due to brief disconnection between the amplifiers and the laptop or computer. Sections containing data loss were marked in 0.5 s segments with 100 ms overlap and excluded from analysis.

Following the application of a channel position montage for both EEG systems, temporal alignment and adjustment of sampling rates were performed. Subsequently, the data from each EEG system was bandpass filtered using 0.1 Hz to 40 Hz finite infinite response filter (FIR). As LSL recorded the data for the cap and grid with slightly differing nominal sampling rates, the data was then resampled to 250 Hz for each, to synchronize the two streams to be merged into one dataset. Identification and interpolation of bad channels of cap data were conducted, and a re-referencing procedure was implemented, referencing the cap data to averaged mastoid electrodes (M1 and M2). Using the spatial approximation approach, computations based on grid channels were performed to obtain virtual 10–20 channels (see vector projections and computations in Figure 1). The grid and cap EEG data were then combined into one dataset and underwent a second round of FIR bandpass filtering, now within the frequency range of 1 to 40 Hz which is optimal for the following Artifact Subspace Reconstruction (ASR) method (refer to Mullen et al., 2015; Plechawska-Wojcik et al., 2019) to remove EEG artifacts.

#### EEG spectra analysis

To compute power spectral density (PSD), preprocessed data was epoched into 10 s epochs from the beginning to the end of the simulation. Epochs that overlap with data loss sections were then removed, as well as any epochs with artifacts, defined by >200 μV peak-to-peak signal amplitude. Epochs were then divided into six 10 min sections, created equally from the beginning to the end of each driving simulation condition, according to where each epoch started. Then we computed the PSD of each section from 1 to 30 Hz, using a multitaper method in the *compute_psd()* function of MNE, and log transferred it to decibel (dB) scale. Data were then compared for the alpha (8–12 Hz), beta (13–26 Hz) and theta (4–8 Hz) bands, separately for cap and trEEGrid channels. For alpha spectra, channel P4_M2 (grid) and channel P4 (cap) were used, as parietal channel alpha has been associated with mental workload (Sciaraffa et al., 2022; Gevins et al., 1997). Beta and theta bands both used channel FPz_M2 (trEEGrid) and Fz (cap), as frontal beta power has been shown to be positively related to mental effort (Pershin et al., 2023) and negatively to vigilance (Sciaraffa et al., 2022), while frontal theta has been associated with mental fatigue (Wascher et al., 2014).

#### Behavioral driving analysis

Behavioral driving data used for analysis included the simulated vehicle's lateral position relative to the center of the right lane, steering angle (angle of the steering wheel in radians) and heading angle (heading angle of the vehicle in degrees with respect to the world). The continuous data from all measures was split into six equal 10-min sections over the whole simulation and for each condition. To assess driving performance, we took the root-mean-squared error (RMSE) lane position, which is defined as the root mean square deviation of the vehicle's lateral position from the center of the lane, in meters.

#### Hazard detection task data

To compute discrimination response times (DRT) to the driving hazard detection task, we subtracted the marker times of the unsafe car appearances from the timing of a valid button press. DRT values were removed if they were < 200 ms, as these fell out of the typical range of the DRT for this particular hazard detection task (~500–2,500 ms) and were likely late responses from the previous event. Across all participants there was only one response faster than 200 ms after stimulus onset.

#### Eye tracking data

The main measure computed from the eye tracking data was percentage closure of the eyes, or PERCLOS (Abe, 2023; Wierwille et al., 1994). PERCLOS was calculated by first extracting information streams for eye openness and validity at each timepoint from both the left and right eye. The openness value was defined by the eye tracking software as the largest sphere that could fit between the lower and upper eyelids (Tobii, Stockholm, Sweden). Validity was marked as a 0/1 value from the eye tracker at each time point to indicate whether the data could be considered valid (1) or invalid (0). An invalid data point indicated some type of error or inability of the eye tracker to properly calculate the openness value (e.g., the participant sneezes or looks away from the simulator). The PERCLOS was then computed by finding the percentage openness relative to the maximum eye openness for each subject, for each eye. Then, PERCLOS was averaged between the left and right eyes, and data points in which both eyes are deemed invalid were removed. PERCLOS calculation was performed with a sliding window over each session, averaging over data with a window length of 60 s, 59 s overlap and 1 s step size.

#### Heart rate variability (HRV)

To extract heart rate variability (HRV) data the continuous ECG data stream for each of the two driving conditions was divided in 1-min intervals. Using a peak finding algorithm based on the function “find_peaks” (SciPy Version 1.7.3; Virtanen et al., 2020) the R-peak of the ECG signal was detected. This was done in a semiautomatic fashion by setting the *prominence* value of said algorithm individually for each subject to ensure accurate peak detection. Heart rate variability was computed first by extracting heart rate (beats-per-minute, BPM) by summing the detected R-peaks in each of the 1-min time windows. We used SDRR in ms as measure of HRV which refers to the standard deviation of the R-to-R peak time difference (ms) of successive peaks for each of the 1-min time window.

#### Psychomotor vigilance task (PVT)

The PVT was implemented in such a way that there was a 1 s response time window and only responses >100 ms were considered valid (Basner et al., 2011; Roach et al., 2006). Valid reaction times were then averaged for each participant and each PVT measurement (baseline, driving session 1, driving session 2).

#### Statistical analysis

The data were analyzed using JASP (JASP Team, 2024). Measures included RMSE lateral position, discrimination response times (DRT), parietal alpha, frontal beta and theta, PERCLOS, HRV, PVT responses, as well as questionnaire responses from the NASA-TLX, SSS, and SSS-Q. For all continuous measures (i.e., RMSE lateral position, DRT, PERCLOS, HRV, parietal alpha, frontal beta and theta), data were averaged over the same six 10 min time sections from the beginning to end of each 60 min scenario, and these 6 values per driving condition were used in further analysis. For ANOVA tests, degrees of freedom and *p*-values are reported using the Greenhouse-Geisser (GG) correction, when applicable. Effect sizes for *p* < 0.05 ANOVA effects are reported using partial eta squared ($\eta_{\text{p}}^{2}$). Upon significant ANOVA tests, *post-hoc* paired *t*-tests were performed, comparing within the factor that reached significance. In the case of multiple *t*-tests, *p*-values are corrected for multiple corrections using the Bonferroni-Holm correction (Abdi, 2010; Holm, 1979).

For each measure, Shapiro-Wilk tests were employed before performing the ANOVA test, to assess normality. In cases of non-normal distribution, such as for the SSS, SSS-Q, PERCLOS and HRV data, non-parametric Friedman tests were carried out separately for each of the two driving modes, if applicable. When significant, the Friedman tests were followed by non-parametric Conover *post-hoc* comparisons tests. When the Friedman test could not be used (i.e., in the case of non-parametrically testing for an interaction), paired Wilcoxon tests were carried out.

For RMSE lane position, a one-factorial (time section T1–T6) repeated-measures ANOVA was used, as this was only possible in the Manual condition. For parietal alpha, frontal beta and theta PSD values, and DRT during driving, 2 (Driving Mode: PAD vs. Manual) × 6 (Time: T1–T6) ANOVAs were used. For PVT response times, a one-factorial ANOVA with three levels (Time: baseline, after the 1st driving session, and after the 2nd driving session) was carried out. For the NASA-TLX, data from all categories (Mental demand, Physical demand, Temporal demand, Performance, Frustration, and Effort) were averaged for all participants (Virtanen et al., 2022) and compared as a paired *t*-test between driving modes (PAD vs. manual).

## Results

For all EEG, driving simulator, eye tracking, and ECG data, one participant was removed due to an error with the photo diode timing correction, leaving 27 subjects for the analyses of these parameters. Additionally, another subject was identified as a statistical outlier and removed from the eye-tracking (PERCLOS) analysis, leaving 26 subjects for the analysis of PERCLOS data.

For the PVT, two subjects were not included due to technical errors in the data collection, leaving 26 subjects for this analysis. For the trEEGrid data, six subjects were removed in analyses which used the FPz_M2 channel, due to excessive noise or disconnection in this channel, leaving 21 subjects for the analysis of frontal theta and beta power in the trEEGrids. Similar issues with bad channels in the cap channels were dealt with by simply removing the channel and interpolating it from neighboring channels. Therefore, for the analyses of the cap data of all 27 participants were included. Channel interpolation is not possible for the trEEGrid system because of the small number of channels, and their distance from each other. For a detailed overview of all statistical results of the *post-hoc* tests we refer to Supplementary Table S1.

### Subjective questionnaires

#### NASA-TLX

**Figure 5:**
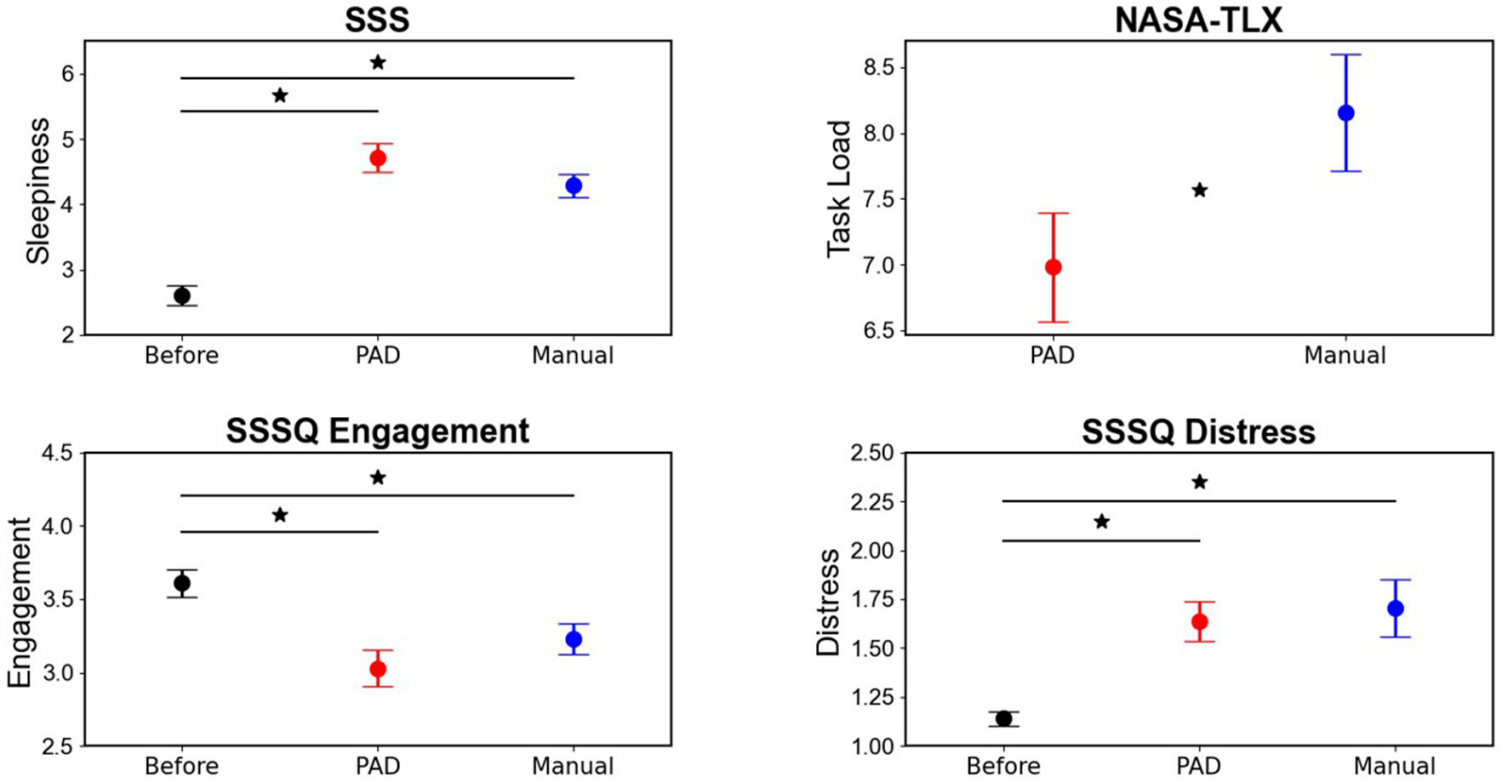
Results of the subjective measures obtained in the study. The NASA-TLX was conducted after each of the two driving conditions (PAD and Manual) but not at baseline as there was no task to be evaluated. The Stanford Sleepiness Scale (SSS) and the Short Stress State Questionnaire (SSS-Q) were administered at baseline (i.e., before driving) and then again after each driving condition (PAD and Manual). Error bars indicate standard error around the mean. *Indicates significant differences between conditions which are at each end of the black bar. Y-axes are adjusted to narrow range for better visualization of effects. The scores for the SSS can range from 1 to 7, for the SSSQ from 1 to 5, and for the NASA-TLX from 0 to 20.

The raw NASA-TLX scores for each of the six subscales were aggregated and then averaged for each participant. Each subscale contained 21 gradations ranging from 0 to 20 (Hart, 1986). Figure 5 depicts the results for the NASA-TLX. Evident from the plots is an increase in task load demand for the Manual compared to the PAD condition. A paired *t*-test revealed a significantly higher task load in the Manual condition (*M* = 8.16) compared to the PAD condition [*M* = 6.98; *M*_*diff*_ = 1.18; *t*(27) = −2.69; *p* = 0.012].

#### Stanford sleepiness scale (SSS)

Figure 5 shows the results for the SSS questionnaire, demonstrating an increase in sleepiness after both conditions compared to the baseline, with slightly higher scores after the PAD than Manual driving mode. A Friedman test showed a significant effect of Mode [χ^2^ (2) = 36.57; *p* < 0.001]. Conover's *post-hoc* comparisons showed that compared to the baseline (*M* = 2.61), a significant increase of sleepiness was found in both the PAD [*M* = 4.71; *M*_*diff*_ = 2.11; *t*(54) = 5.73; *p* < 0.001] and Manual condition [*M* = 4.29; *M*_*diff*_ = 1.68; *t*(54) = 4.58; *p* < 0.001]. However, the difference in sleepiness between PAD and Manual was not significant [*M*_*diff*_ = 0.43; *t*(54) = 1.15; *p* = 0.26]. A score of three corresponds to “Neutral, okay, neither sleepy nor alert,” a score of four to “Somewhat sleepy, not fully alert” and five to “Sleepy, but no difficulty staying awake.”

#### Short stress state questionnaire SSS-Q

Figure 5 depicts the results from the SSS-Q for engagement, showing the lowest engagement in the PAD mode, followed by Manual. For engagement, a Friedman test showed a significant effect of Mode [χ^2^ (2) = 16.71; *p* < 0.001]. Conover's *post-hoc* comparisons showed that compared to the baseline (*M* = 3.61) a significant decrease in engagement for both PAD [*M* = 3.03; *M*_*diff*_ = 0.58; *t*(54) = 4.08; *p* < 0.001] and Manual [*M* = 3.23; *M*_*diff*_ = 0.38; *t*(54) = 2.35; *p* = 0.045]. Although the level of engagement was lower in the PAD compared to the Manual driving mode, the difference did not reach statistical significance [*M*_*diff*_ = 0.2; *t*(54) = 1.73; *p* = 0.09].

Figure 5 depicts the results from the SSS-Q for distress, showing an increase in level of distress after both PAD and Manual driving relative to the baseline measurement. For engagement, a Friedman test showed a significant effect of Driving mode [χ^2^ (2) = 23.53; *p* < 0.001]. Conover's *post-hoc* comparisons showed that compared to the baseline (*M* = 1.14) a significant increase in distress for both PAD [*M* = 1.64; *M*_*diff*_ = 0.5; *t*(54) = 4.18; *p* < 0.001] and Manual [*M* = 1.7; *M*_*diff*_ = 0.56; *t*(54) = 4.25; *p* < 0.001] but no difference between the two driving modes [*M*_*diff*_ = 0.06; *t*(54) = 0.07; *p* = 0.94].

### Driving simulator measures

#### RMSE lateral position

**Figure 6:**
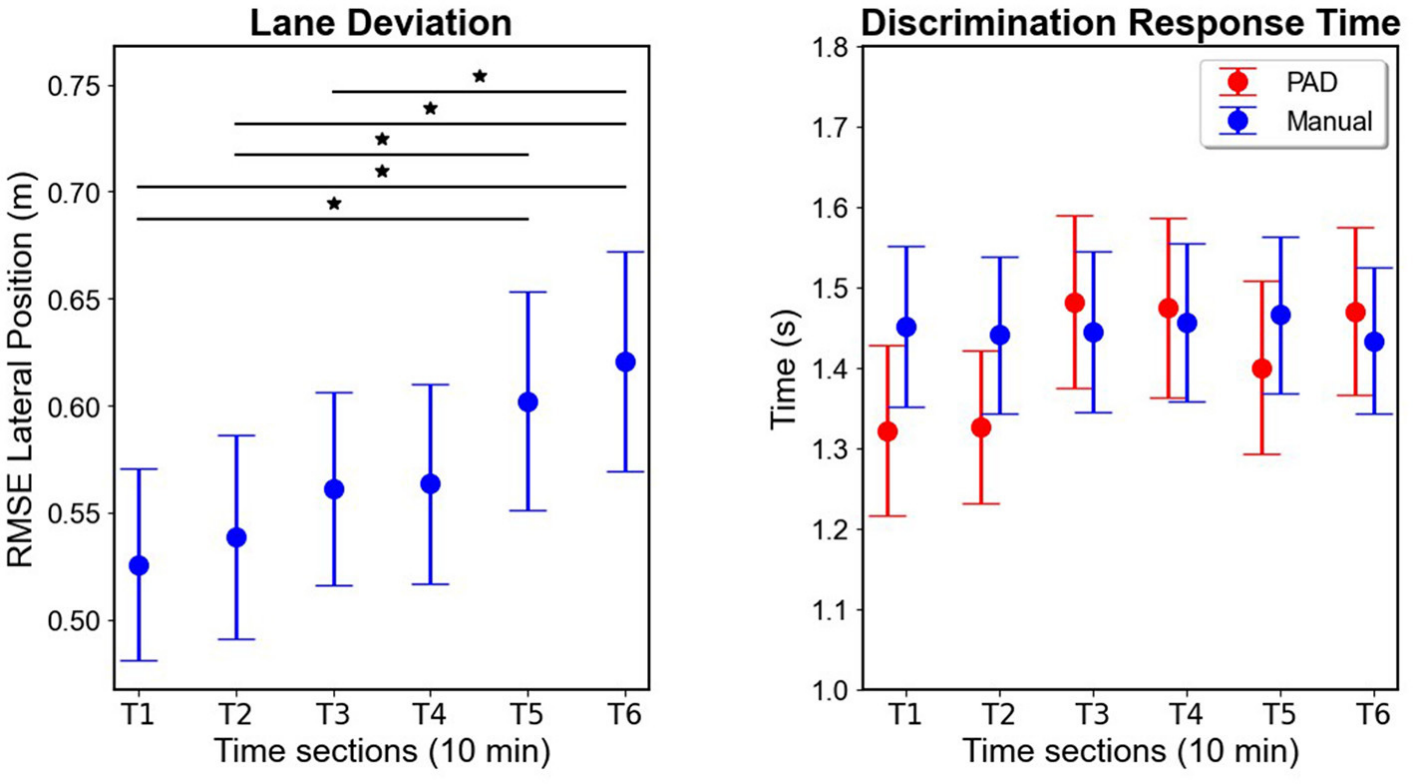
Driving simulator measures. **(Left)**: RMSE lateral positions over time in the Manual condition. **(Right)**: Mean discrimination response time (DRT) to the hazard detection task during driving in both conditions. Error bars indicate standard error of the mean. *Indicate significant differences between conditions which are at each end of the black bar. Y-axes are adjusted to the relevant range of the actual data for better visualization of effects.

RMSE lane position (in meters) relative to the center of the right-hand lane in the Manual condition is plotted in Figure 6. In the PAD condition lane keeping was controlled automatically and is therefore not plotted nor analyzed. In the Manual condition RMSE lateral lane position increased gradually over time reflected in a significant effect of Time [*F*_(2.14, 55.64)_ = 6.67; *p* = 0.002; $\eta_{p}^{2}$ = 0.2]. *Post-hoc* paired *t*-tests indicated significantly higher RMSE lane position at T5 (*M* = 0.60 m) than T1 [*M* = 0.53 m; *M*_*diff*_ = 0.076 m; *t*(26) = 3.83; *p* = 0.003] and T2 [*M* = 0.54 m; *M*_*diff*_ = 0.063 m; *t*(26) = 3.18; *p* = 0.022]. Additionally, T6 (*M* = 0.62 m) showed significantly higher RMSE lane position than T1 [*M* = 0.53 m; *M*_*diff*_ = 0.095 m; *t*(26) = 4.75; *p* < 0.001], T2 [*M* = 0.54 m; *M*_*diff*_ = 0.082 m; *t*(26) = 4.1; *p* = 0.001], and T3 [*M* = 0.56 m; *M*_*diff*_ = 0.059 m; *t*(26) = 2.98; *p* = 0.038].

#### Driving hazard detection task (DRT and accuracy)

Driving hazard detection task DRT (in seconds) is plotted in Figure 6. Evident from the plot is relatively constant DRT throughout the hour in the Manual mode. In PAD mode, DRT is faster at the beginning and then slows down to a similar level to the Manual condition around T3 and later. Here we found no significant main effects of Mode [*F*_(1, 26)_ = 0.66; *p* = 0.42], or Time [*F*_(3.65, 94.8)_ = 1.78; *p* = 0.15], but a significant interaction effect [*F*_(3.66, 95.03)_ = 2.58; *p* = 0.047; $\eta_{p}^{2}$ = 0.09]. As seen in Figure 6 DRT is faster for PAD than Manual in T1 and T2 but *post-hoc* paired *t*-tests corrected for multiple comparisons did not find any significant differences between any condition in any 10-min time section (all *p*'s > 0.1). Response accuracy was equally high in both the Manual (*M* = 98.89%, *SD* = 1.97) and the PAD condition (*M* = 98.78%, *SD* = 2.74) indicating that participants were able to distinguish both types of parked cars and do the task well.

#### PERCLOS

**Figure 7:**
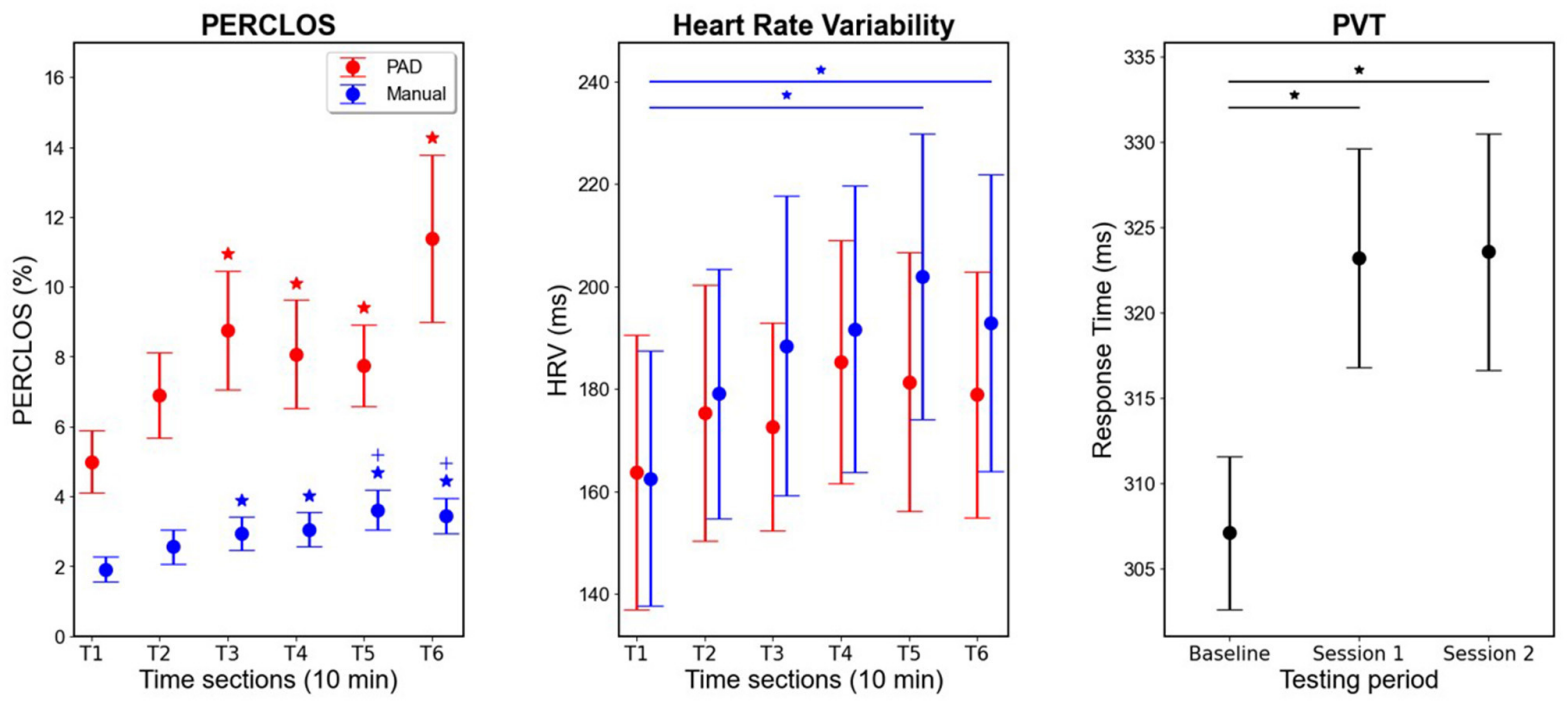
**(Left)**: Mean PERCLOS for PAD and Manual driving conditions. PERCLOS values range between 0 and 100%. Colored * indicate significant differences from T1 in the respective condition. Colored + indicate significant difference from T2 in the respective condition. **(Middle)**: Mean HRV for PAD and Manual driving conditions. *Indicate significant differences between conditions which are at each end of the bar, with colors corresponding to condition. **(Right)**: Mean PVT response times at baseline, after the 1^st^ driving session and after the 2^nd^ driving session. * indicate significant differences between conditions which are at each end of the black bar. Driving conditions were counterbalanced. Error bars indicate standard error of the mean. Y-axes are adjusted to the relevant range of the actual data for better visualization of effects.

PERCLOS data (in percentage) is plotted in Figure 7. Evident from the plot is much higher values in the PAD than Manual mode, and an increase for both over time. A main effect for Time was found in both the PAD [χ^2^ (5) = 27.8; *p* < 0.001] and Manual modes [χ^2^ (5) = 51.85; *p* < 0.001]. Conover *post hoc* tests between times in the PAD condition indicated significant increases from T1 (*M* = 0.05) to T3 [*M* = 0.088; *M*_*diff*_ = 0.038; *t*(125) = 4.33; *p* < 0.001], T4 [*M* = 0.081; *M*_*diff*_ = 0.031; *t*(125) = 3.38; *p* = 0.012], T5 [*M* = 0.077; *M*_*diff*_ = 0.028; *t*(125) = 3.45; *p* = 0.01], and T6 [*M* = 0.11; *M*_*diff*_ = 0.064; *t*(125) = 4.48; *p* < 0.001]. Conover *post-hoc* tests between times in the Manual condition indicated significant increases from T1 (*M* = 0.019) to T3 [*M* = 0.029; *M*_*diff*_ = 0.01; *t*(125) = 3.67; *p* = 0.004], T4 [*M* = 0.031; *M*_*diff*_ = 0.011; *t*(125) = 3.67; *p* = 0.004], T5 [*M* = 0.036; *M*_*diff*_ = 0.017; *t*(125) = 5.21; *p* < 0.001] and T6 [*M* = 0.034; *M*_*diff*_ = 0.015; *t*(125) = 6.09; *p* < 0.001], as well as from T2 (*M* = 0.026) to T5 [*M*_*diff*_ = 0.011; *t*(125) = 3.6; *p* = 0.005] and T6 [*M*_*diff*_ = 0.009; *t*(125) = 4.48; *p* < 0.001]. To check the interaction, we carried out paired Wilcoxon tests between PAD and Manual at each time, finding significantly higher PERCLOS in PAD in each time section [T1: *M*_*diff*_ = 0.031; *t*(25) = 3.87; *p* < 0.001; T2: *M*_*diff*_ = 0.043; *t*(25) = 3.89; *p* < 0.001; T3: *M*_*diff*_ = 0.059; *t*(25) = 3.73; *p* < 0.001; T4: *M*_*diff*_ = 0.05; *t*(25) = 3.58; *p* < 0.001; T5: *M*_*diff*_ = 0.041; *t*(25) = 3.89; *p* < 0.001; T6: *M*_*diff*_ = 0.08; *t*(25) = 3.56; *p* < 0.001].

#### Heart rate variability (HRV)

Figure 7 shows HRV (in milliseconds) during the driving tasks. Evident from the plot is an increase over time for both conditions, more so for the Manual than PAD condition. The data was found to be non-normally distributed, therefore Friedman tests indicated a significant effect of time in the Manual condition [χ^2^ (5) = 14.39; *p* = 0.013] but not in the PAD condition [χ^2^ (5) = 9.52; *p* = 0.09]. Conover *post-hoc* comparisons for the Manual condition indicated significantly higher HRV in T5 [*M* = 201.93; *M*_*diff*_ = 39.38; *t*(130) = 3.31; *p* = 0.018], and T6 [*M* = 192.86; *M*_*diff*_ = 30.31; *t*(130) = 3.03; *p* = 0.042] compared to T1 (*M* = 162.55).

#### Psychomotor vigilance task (PVT)

Figure 7 depicts the RT results of the PVT task (in milliseconds). Evident from the plot is an increase in RT after the first and second hour of driving compared to the baseline measurement. This is confirmed by a significant main effect of Time [*F*_(2, 50)_ = 7.92; *p* = 0.001; $\eta_{p}^{2}$ = 0.24]. *Post-hoc* tests indicated increased response times relative to the baseline PVT (*M* = 307.12 ms) after the first 1 h driving session [averaged across Manual and PAD; *M* = 323.2 ms; *M*_*diff*_ = 16.07 ms; *t*(25) = −3.41; *p* = 0.003] as well as after the second 1h driving session [averaged across Manual and PAD; *M* = 323.57 ms; *M*_*diff*_ = 16.44 ms; *t*(25) = −3.49; *p* = 0.003]. However, there was no significant difference between response times after the first and second driving session [*M*_*diff*_ = 0.37 ms; *t*(25) = −0.078; *p* = 0.94].

### EEG spectra

#### Parietal alpha

**Figure 8:**
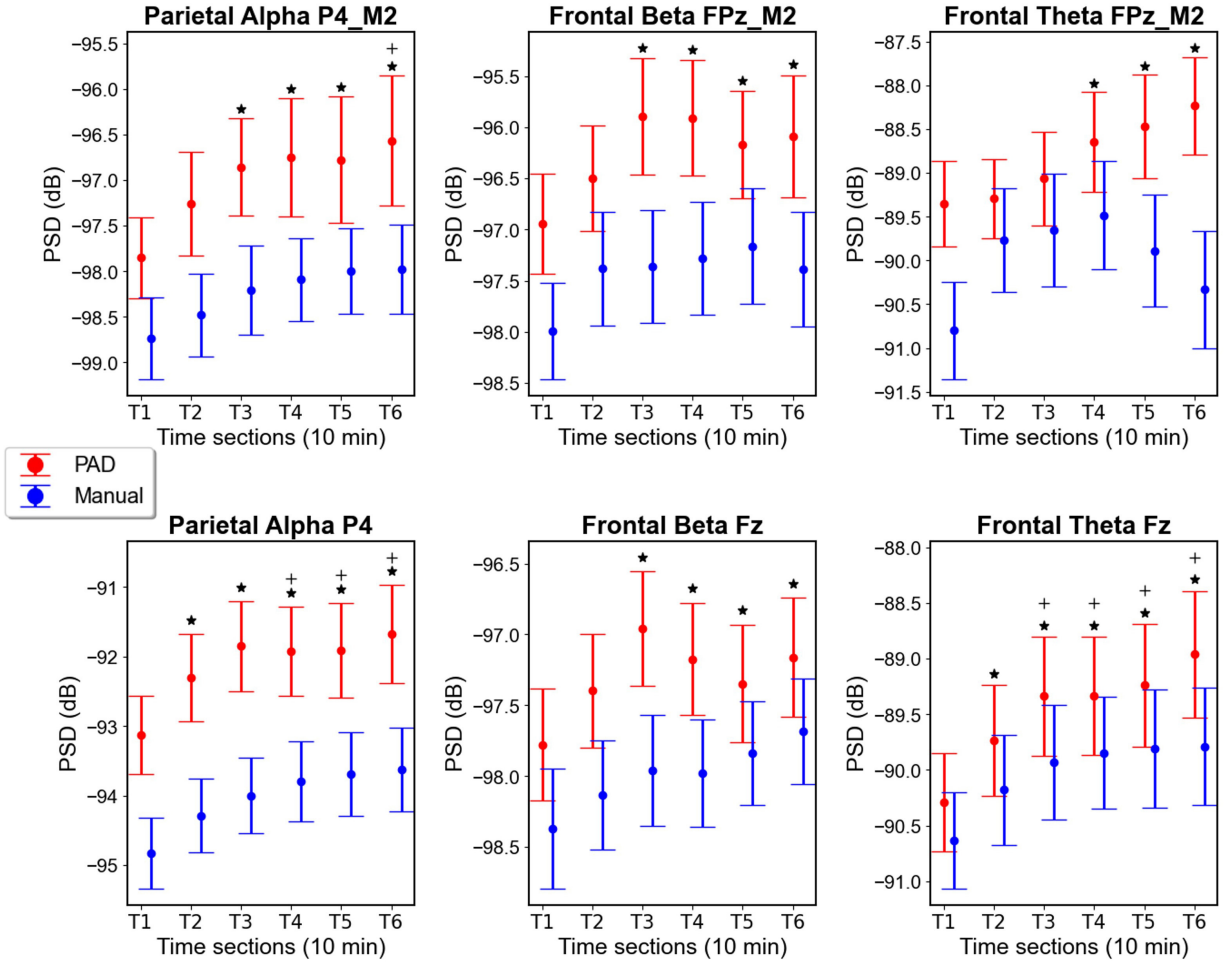
PSD plots for mean alpha, beta and theta power [in (V^2^)/Hz converted to dB] with error bars indicating standard error in the measured electrode sites for both grid data **(top)** and cap data **(bottom)**; *Indicate significant differences from T1 in both conditions [PAD (red) and Manual (blue)] combined. + indicate significant differences from T2 in both conditions combined.

Parietal alpha power [in (V^2^)/Hz converted to dB] for the grids in channel P4_M2 is plotted in Figure 8. Evident from the plot is that parietal alpha is higher in the PAD condition than the Manual condition. Additionally, parietal alpha increases over time within both conditions. ANOVA tests confirmed this, as there was a significant main effect of both Mode [*F*_(1, 26)_ = 19.29; *p* < 0.001; $\eta_{p}^{2}$ = 0.23], and Time [*F*_(1.59, 41.25)_ = 8.8; *p* = 0.001; $\eta_{p}^{2}$ = 0.25], but no interaction effect [*F*_(1.89, 49.25)_ = 0.73; *p* = 0.48; $\eta_{p}^{2}$ = 0.005]. A *post-hoc t*-test between the grid PAD and Manual (*M* = −98.25 dB) alpha power averaged over time indicate significantly higher alpha power in the PAD condition [*M* = −97.01 dB; *M*_*diff*_ = 1.24; *t*(26) = 4.39; *p* < 0.001]. *Post-hoc* paired *t*-tests comparing each time section to each other, while averaging over mode, indicate significant alpha power increases from T1 (*M* = −98.29 dB) to T3 [*M* = −97.53 dB; *M*_*diff*_ = 0.76 dB; *t*(26) = −4.15; *p* = < 0.001], T4 [*M* = −97.42 dB; *M*_*diff*_ = 0.87 dB; *t*(26) = −4.76; *p* < 0.001], T5 [*M* = −97.39 dB; *M*_*diff*_ = 0.91 dB; *t*(26) = −4.95; *p* < 0.001], and T6 [*M* = −97.27 dB; *M*_*diff*_ = 1.02 dB; *t*(26) = −5.57; *p* < 0.001]. Additionally, there was a significant difference in average alpha power between T2 (*M* = −97.87 dB) and T6 [*M* = −97.27 dB; *M*_*diff*_ = 0.6 dB; *t*(26) = −3.27; *p* = 0.015]. These differences demonstrate, as can be seen in the plots, a sharp increase at the beginning of the scenario, which plateaus toward the middle and end of the scenario.

In the cap data, the same effects can be seen in Figure 8 for parietal alpha from channel P4, with higher power in the PAD condition and an increase over time for both conditions. A significant main effect was found for Mode [*F*_(1, 26)_ = 66.33; *p* < 0.001; $\eta_{p}^{2}$ = 0.72] and Time [*F*_(2.5, 64.97)_ = 24.13; *p* < 0.001; $\eta_{p}^{2}$ = 0.48], but no interaction [*F*_(4.04, 105.11)_ = 0.96; *p* = 0.48]. A *post-hoc t*-test between the PAD and Manual (*M* = −94.04 dB) alpha power averaged over time indicates significantly higher alpha power in the PAD condition [*M* = −92.13 dB; *M*_*diff*_ = 1.91 dB; *t*(26) = 8.14; *p* < 0.001]. *Post-hoc* paired *t*-tests averaging over mode and comparing each time section to each other indicate significant alpha power increases from T1 (*M* = −93.98 dB) to T2 [*M* = −93.3 dB; *M*_*diff*_ = 0.68 dB; *t*(26) = −4.86; *p* < 0.001], T3 [*M* = −92.93 dB; *M*_*diff*_ = 1.05 dB; *t*(26) = −7.5; *p* < 0.001], T4 [*M* = −92.86 dB; *M*_*diff*_ = 1.12; *t*(26) = −7.96; *p* < 0.001], T5 [*M* = −92.80 dB; *M*_*diff*_ = 1.18 dB; *t*(26) = −8.39; *p* < 0.001] and T6 [*M* = −92.65 dB; *M*_*diff*_ = 1.33 dB; *t*(26) = −9.47 *p* < 0.001], as well as from T2 to T4 [*M*_*diff*_ = 0.43 dB; *t*(26) = −3.09; *p* = 0.019], T5 [*M*_*diff*_ = 0.5 dB; *t*(26) = −3.53; *p* = 0.005], and T6 [*M*_*diff*_ = 0.65 dB; *t*(26) = −4.6; *p* = 0.001].

#### Frontal beta

Figure 8 depicts frontal beta power [in (V^2^)/Hz converted to dB] in the grid FPz_M2 channel. Evident from the plot is significantly higher beta power in the PAD condition compared to Manual driving, while both driving modes appear to increase over time. Here we found a significant main effect of Mode [*F*_(1, 20)_ = 13.34; *p* = 0.002; $\eta_{p}^{2}$ = 0.4] and Time [*F*_(2.5, 50.1)_ = 4.87; *p* = 0.007; $\eta_{p}^{2}$ = 0.196], but no interaction [*F*_(2.94, 58.78)_ = 0.65; *p* = 0.58, $\eta_{p}^{2}$ = 0.031]. A *post-hoc t*-test between the PAD and Manual (*M* = −97.43 dB) beta power averaged over time indicated significantly higher beta power in the PAD condition [*M* = −96.25 dB; *M*_*diff*_ = 1.18 dB; *t*(20) = 3.65; *p* = 0.002]. *Post-hoc* comparisons of time sections, averaged over Mode, indicate significant increases in beta power from T1 (*M* = −97.47 dB) to T3 [*M* = −96.63 dB; *M*_*diff*_ = 0.84 dB; *t*(20) = −3.95; *p* = 0.002], T4 [*M* = −96.59 dB; *M*_*diff*_ = 0.88 dB; *t*(20) = −4.12; *p* = 0.001], T5 [*M* = −96.67 dB; *M*_*diff*_ = 0.80 dB; *t*(20) = −3.77; *p* = 0.004], and T6 [*M* = −96.74; *M*_*diff*_ = 0.73 dB; *t*(20) = −3.42; *p* = 0.011].

In the beta power [in (V^2^)/Hz converted to dB] cap data at channel Fz, similar effects can be seen at Figure 8. A significant main effect was found for Mode (*F*(1,26) = 10.07; *p* = 0.004; $\eta_{p}^{2}$ = 0.28) and Time [*F*_(3.39, 88.18)_ = 9.12; *p* < 0.001; $\eta_{p}^{2}$ =0.26], but there was no significant interaction [*F*_(3.11, 80.82)_ = 1.25; *p* = 0.3; $\eta_{p}^{2}$ = 0.046]. *Post-hoc* tests in which we averaged over all time sections for each mode found a significant difference between PAD (*M* = −97.3 dB) and Manual driving beta power [*M* = −98.0 dB; *M*_*diff*_ = 0.69; *t*(26) = 3.17; *p* = 0.004]. *Post-hoc* comparisons of time sections which were averaged over mode, indicate significant increases in beta power from T1 (*M* = −98.07 dB) to T3 [*M* = −97.46 dB; *M*_*diff*_ = 0.61 dB; *t*(26) = −5.46; *p* < 0.001], T4 [*M* = −97.58 dB; *M*_*diff*_ = 0.5 dB; *t*(26) = −4.41 *p* < 0.001], T5 [*M* = −97.59 dB; *M*_*diff*_ = 0.48 dB; *t*(26) = −4.26; *p* < 0.001], and T6 [*M* = −97.42 dB; *M*_*diff*_ = 0.65 dB; *t*(26) = −5.77; *p* < 0.001], and also from T2 to T6 [*M*_*diff*_ = 0.34; *t*(26) = −3.04; *p* = 0.032].

#### Frontal theta

Figure 8 depicts theta power [in (V^2^)/Hz converted to dB] in the FPz_M2 channel. Evident from the plot is higher theta power in the PAD than Manual condition, with the power increasing with time in the PAD condition, and an inverted “U” shape in the Manual condition. Here we found a significant main effect of Mode [*F*_(1, 20)_ = 8.67; *p* = 0.008; $\eta_{p}^{2}$ = 0.302], and Time [*F*_(2.15, 42.97)_ = 3.93; *p* = 0.025; $\eta_{p}^{2}$ = 0.16], as well as a significant interaction effect [*F*_(2.53, 50.57)_ = 3.88; *p* = 0.019; $\eta_{p}^{2}$ = 0.16]. A *post-hoc* test comparing the PAD to Manual driving, averaged over all time sections showed a significant difference between PAD (*M* = −88.84 dB) and Manual [*M* = −89.99 dB; *M*_*diff*_ = 1.14; *t*(20) = 2.94; *p* = 0.008]. *Post-hoc* comparisons of time sections averaged over driving mode showed a significant increase from T1 (*M* = −90.08 dB) to T4 [*M* = −89.07 dB; *M*_*diff*_ = 1.01 dB; *t*(20) = −3.94; *p* = 0.002], T5 [*M* = −89.18 dB; *M*_*diff*_ = 0.90 dB; *t*(20) = −3.49; *p* = 0.01], and T6 [*M* = 89.28 dB; *M*_*diff*_ = 0.79 dB; *t*(20) = −3.09; *p* = 0.034]. However, these effects may be superseded by the interaction effect. Therefore, we conducted another paired comparison between all modes and time sections. Here we found a significant increase the Manual condition T1 (*M* = −90.80 dB) to T4 (*M* = −89.48 dB; *M*_*diff*_ = 1.32 dB; −3.88; *p* = 0.009). No significant differences were found between time sections within the PAD condition (all p's > 0.05), but PAD (*M* = −88.23 dB) was significantly higher than Manual (*M* = −90.33 dB) mode in T6 [*M*_*diff*_ = 2.09 dB; *t*(20) = 4.34; *p* = 0.005].

In the theta power [in (V^2^)/Hz converted to dB] cap data at channel Fz, similar effects to the grid can be observed. PAD appears to have higher theta power than the Manual condition. Both modes increase at the beginning, with the PAD mode continuing to increase while the Manual mode shows a plateau after T3. Here we found a significant main effect of both Mode [*F*_(1, 26)_ = 7.93; *p* = 0.009; $\eta_{p}^{2}$ = 0.23], and Time [*F*_(2.43, 63.26)_ = 23.97; *p* < 0.001; $\eta_{p}^{2}$ = 0.48], but no interaction [*F*_(3.13, 81.25)_ = 1.57; *p* = 0.2; $\eta_{p}^{2}$ = 0.057]. A *post-hoc* test comparing the theta power in PAD to Manual driving, averaged over all time sections, showed a significantly higher power in the PAD (*M* = −89.48 dB) than Manual [*M* = −90.03 dB; *M*_*diff*_ = 0.55 dB; *t*_(26)_ = 2.82; *p* = 0.009] mode. Another *post-hoc* test between time sections and averaging over mode found significant increases from T1(*M* = −90.46 dB) to T2 [*M* = −89.96 dB; *M*_*diff*_ = 0.50 dB; *t*(26) = −4.41; *p* < 0.001], T3 [*M* = −89.63 dB; *M*_*diff*_ = 0.83 dB; *t*(26) = −7.24; *p* < 0.001], T4 [*M* = −89.59 dB; *M*_*diff*_ = 0.87 dB; *t*(26) = −7.63; *p* < 0.001], T5 [*M* = −89.52 dB; *M*_*diff*_ = 0.44; *t*(26) = −8.22; *p* < 0.001] and T6 [*M* = −89.37; *M*_*diff*_ = 1.09 dB; *t*(26) = −9.53; *p* < 0.001]. Additionally, significant increases were found from T2 to T3 [*M*_*diff*_ = 0.32 dB; *t*(26) = −2.84; *p* = 0.037], T4[*M*_*diff*_ = 0.37 dB; *t*(26) = −3.22; *p* = 0.013], T5 [*M*_*diff*_ = 0.44 dB; *t*(26) = −3.82; *p* = 0.002], and T6 [*M*_*diff*_ = 0.59 dB; *t*(26) = −5.12; *p* < 0.001].

Further analyses regarding theta power in cap electrodes FP1 and FP2 are shown in Supplementary Figure S1 to indicate an inverted “U” shape in the Manual condition similar to the results of the FPz_M2 channel of the trEEGrid.

#### Correlational analyses EEG and driving performance

**Figure 9:**
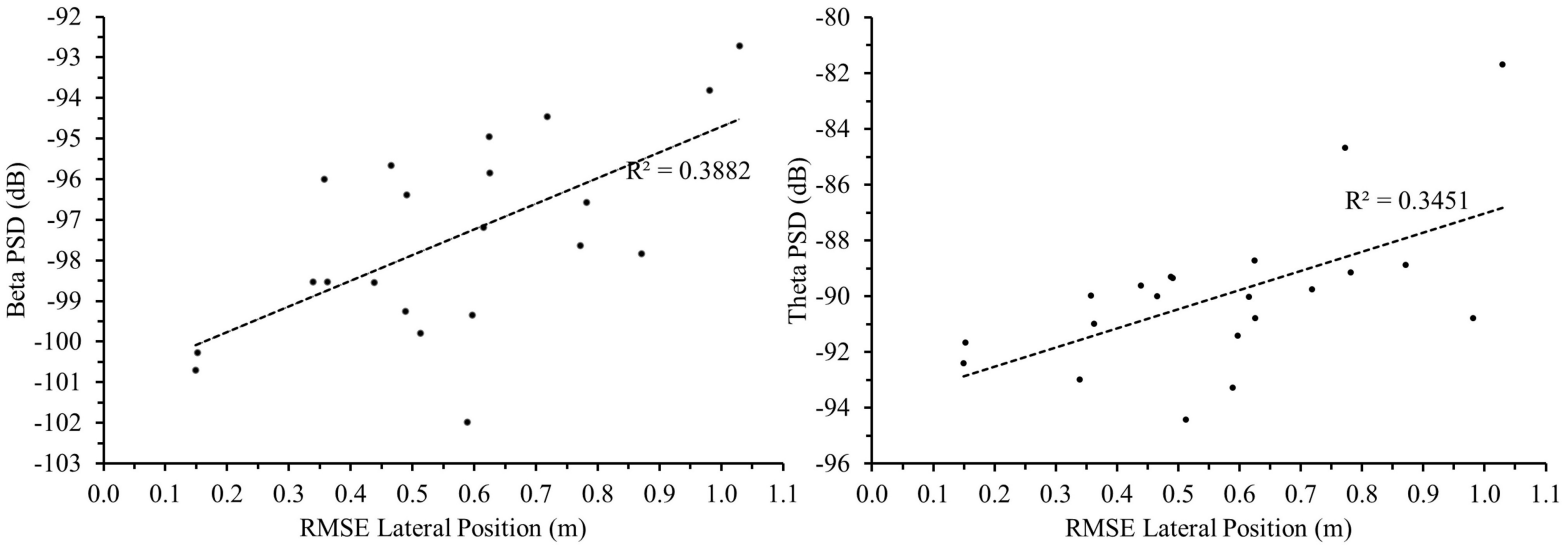
Scatterplots (*N* = 21) visualizing the correlation between beta PSD **(left)** and theta PSD **(right)** in [(V^2^)/Hz dB] at electrode FPz_M2 (trEEGrid) with RMSE Lateral Position (m) for average values across all six time sections.

To further our understanding of the relationship between brain activity and behavior, we were interested in the association between Manual driving and EEG recorded with the trEEGrid. Therefore, we computed correlations between individual RMSE lateral position (index of driving performance) and the PSD values for alpha (electrode P4_M2), beta (electrode FPz_M2) and theta (electrode FPz_M2). The Pearson r correlation values are listed in Table 1. Scatterplots for the correlation between the average RMSE lateral position across all 6 time-sections and average beta and theta are depicted in Figure 9. The results show that individuals with higher beta and theta PSD values also show higher deviations from the center of the lane.

**Table 1:** Pearson *r* values and corresponding *p*-values of the correlation between EEG PSD values (alpha, beta, theta) recorded with the trEEGrid and RMSE of the lateral position (LatPos) as index for driving performance for each time section (T1–T6) as well as the average across all time sections.

**Correlation variables**	**Alpha**		**Beta**		**Theta**	
	***r(25)***	***p***	***r(19)***	***p***	***r(19)***	***p***
EEG_T1 & LatPos_T1	0.346	0.077	0.432	0.05^*^	0.595	0.004^*^
EEG_T2 & LatPos_T2	0.331	0.092	0.618	0.003^*^	0.544	0.011^*^
EEG_T3 & LatPos_T3	0.342	0.081	0.597	0.004^*^	0.553	0.009^*^
EEG_T4 & LatPos_T4	0.376	0.053	0.537	0.012^*^	0.464	0.034^*^
EEG_T5 & LatPos_T5	0.335	0.088	0.610	0.003^*^	0.564	< 0.008^*^
EEG_T6 & LatPos_T6	0.370	0.058	0.563	0.008^*^	0.516	< 0.017^*^
EEG_avgT1T6 & LatPos_avgT1T6	0.356	0.068	0.623	0.003^*^	0.587	< 0.005^*^

^*^p-value < 0.05.

## Discussion

In this study, participants were asked to drive two 1-h scenarios in a driving simulator, one in which they were manually controlling the vehicle and one in which they monitored while the vehicle was autonomously controlled. Participants also performed a concurrent driving-relevant stimulus detection task and wore both a full-cap EEG system and a novel, mobile EEG-electrode-grid system. To our knowledge, this is the first study that investigates the viability of a reduced channel EEG grid sensor system (trEEGrid) in measuring vigilance during simulated driving, compared to a full-cap system recording EEG simultaneously. The results from both EEG systems indicate EEG-power increases in fatigue and vigilance-related EEG frequency bands during PAD compared to manual driving, as well as increases over time. These differences coincide with the same pattern in physiological measures of fatigue (PERCLOS) and decreased subjective ratings of task-load (NASA-TLX) during PAD. Further, brain activity results from both the cap and trEEGrid showed significant changes earlier than other measures of vigilance and fatigue, indicating the potential for spectral EEG as a more effective measure of vigilance during driving. In addition to further our understanding of EEG parameters as important markers of vigilance and changes thereof, the results underline that the novel trEEGrid system can provide high-quality EEG data comparable to traditional 10–20 EEG-electrodes. From a neuroergonomics perspective this is of relevance as it brings the methodology of EEG a step closer to real-world application.

### Subjective questionnaires

In general, results from the questionnaires indicated that our tasks were successful in inducing a monotonous and fatiguing driving scenario thereby taxing the participants' level of vigilance. The SSS and SSSQ scales showed that participants were significantly more fatigued, less engaged, and experiencing more distress after both the PAD and Manual scenarios, compared to before the scenarios. However, these measures did not show significant differences between PAD and manual driving. While subjective measures like the SSS are generally effective in detecting sleepiness during driving (Cai et al., 2021), they can be subject to bias as participants are required to remember their mental state during the drive (McWilliams and Ward, 2021). Additional information gathered from PERCLOS during the scenarios indicates that participants were likely experiencing more physiological fatigue in the PAD compared to the Manual condition. Furthermore, the NASA-TLX, found significantly lower task load for the PAD condition, which can lead to reduced task vigilance and fatigue through cognitive underload (McWilliams and Ward, 2021; Thomson et al., 2015).

### Driving measures

RMSE lateral position increased over time, similar to previous findings in driving simulators (van der Hulst et al., 2001; Ting et al., 2008; Thiffault and Bergeron, 2003) and on-road driving (Verster and Roth, 2013). This is likely due to reductions in vigilance toward the driving task as individuals tend to become more fatigued over time and experience vigilance decrement as they start to deviate from the center of the driving lane (Philip et al., 2005; Verster and Roth, 2013).

No significant main effect of Time or Mode was found in DRT of the concurrent hazard detection task, contrary to some other dual-task driving studies, which found decreases in task performance over time, especially in the PAD condition (Greenlee et al., 2018, 2019, 2022). However, the significant interaction effect indicates that our task effects did differentially change over time between the Manual and PAD condition, with the DRT increasing slightly over time for the PAD condition, while staying relatively stable in the Manual condition. This result is similar to previous results found by Greenlee et al. (2022) in the false alarms and response sensitivity patterns in which responses to the PAD condition showed performance decrements over time while the Manual driving condition did not. This is consistent with the Manual task being more engaging, promoting a stable level of attention for the participants to perform the task. Meanwhile, participants responded faster in the first 2 time periods (i.e., 20 min) of the PAD condition, likely due to the decreased workload required to perform the driving task, allowing increased resources toward the task. However, this benefit is not significantly different from the Manual condition within these two time periods, and any benefit dissipates after 20 min, likely due to the fatigue and reduced vigilance over time associated with PAD driving (Greenlee et al., 2018, 2019, 2022; Körber et al., 2015a,b; Mok et al., 2015).

Our results for the concurrent hazard detection task were not as clear or pronounced, compared to previous driving simulator vigilance tasks (Greenlee et al., 2018, 2019, 2022), with no main effects and no significant *post-hoc* tests. This is possibly due to the less challenging nature of our hazard detection task. In our task, the roadside vehicles were visible until the driver's vehicle passed them, mimicking how this obstacle would appear in real life. This made the task more realistic but also gave the participant a clearer view of the vehicle as they drove closer, making the task easier. This is also evidenced by somewhat slower DRT in our study than in a similar previous study, in which the participants could only see a simulated hazardous car for 200 ms (Greenlee et al., 2018). Additionally, performance accuracy was close to ceiling with an error rate of under 2% in both conditions, making it too low to reliably compare, but indicating that the task was not particularly difficult. It appears that vigilance tasks during driving are sensitive to difficulty, as other studies with relatively easy vigilance tasks have also not found time-on-task effects in DRT (Körber et al., 2015a,b), in some cases while also showing physiological signs of fatigue (Körber et al., 2015a).

### PERCLOS

As expected, PERCLOS values increased over time in both conditions, and were significantly higher in the PAD condition than during Manual driving. PERCLOS difference in driving mode indicates that even though we did not see a significant difference in the SSS questionnaire between the two driving modes, there is likely an increase in drowsiness during PAD compared to Manual driving that the participants may not have had full awareness of. The effect of driving mode is also consistent with previous literature, as PERCLOS has also been shown to increase during various fatigue-inducing driving scenarios, such as nighttime driving and after sleep deprivation (for a review, see Abe, 2023). The time-on-task effects were also not surprising, as PERCLOS has been shown to increase over time during PAD (Heikoop et al., 2017; Jarosch et al., 2019), as well as in Manual driving (Golz et al., 2010). The range of PERCLOS values may have also been somewhat smaller than in some other studies (e.g., Arefnezhad et al., 2022; Abe, 2023), as our participants were rested, while a lot of studies on drowsiness during driving induced this drowsiness through some type of sleep deprivation.

### EEG spectra: effects of driving mode

In both EEG systems, PSD values of parietal alpha, and frontal theta and beta were all higher during PAD compared to Manual driving, throughout the driving task. This is likely due to the reduced engagement required to perform automated driving, which can result in cognitive underload, leading to fatigue and mind-wandering, and therefore reduced vigilance toward the driving task (McWilliams and Ward, 2021). Alpha power has been shown to increase with reduced attentional processing, as well as during lapses in attention toward external stimuli (Borghini et al., 2014; Klimesch, 2012; McWilliams and Ward, 2021). Parietal alpha has been shown previously to increase during PAD and has been used as a measure of vigilance decrement during PAD tasks (Cisler et al., 2019). Both parietal channel alpha and frontal channel beta power have been observed to correlate negatively with vigilance and attention (Sciaraffa et al., 2022), and frontal beta in particular has been correlated with decrements in vigilance (Molina et al., 2013). Additionally, alpha and theta power have been shown to enhance during mind-wandering (Compton et al., 2019; Da Silva et al., 2022), which is much more likely to be happening during PAD.

### EEG spectra: time on task

In both EEG systems we found significant main effects of time in our measures of parietal channel alpha as well as frontal channel theta and beta. This was to be expected, as increases over time have been shown for alpha during manual (Craig et al., 2012; Simon et al., 2011) and PAD driving tasks (Cisler et al., 2019), as well as for theta during manual driving (Arefnezhad et al., 2022; Awais et al., 2014; Craig et al., 2012). Alpha and theta power have been shown to increase with time on task (Wascher et al., 2014; Tran et al., 2020) often marking the transition from wakefulness to drowsiness (Awais et al., 2014; Stancin et al., 2021). Theta power has been shown to increase in relation to increasing PERCLOS during a driving task, with the relationship strong enough to use EEG to predict PERCLOS levels (Arefnezhad et al., 2022). Theta increases over time in cognitive tasks have been interpreted as being related to increased mental effort to maintain task performance over time (Wascher et al., 2014).

One unexpected finding was an interaction effect between Mode and Time for theta power in the frontal grid channel (FPz_M2), but not in the cap Fz channel. This may highlight a slight difference in the information collected between the trEEGrid and cap data, as the FPz_M2 channel of the trEEGrid is located lower and more frontal on the forehead than the Fz channel of the cap. In the grid data, theta power increased over time for the PAD condition but appeared to decrease toward the end of the task for the Manual condition. A similar change in power values over time in the Manual condition was also observed for the cap channels FP1 and FP2, which are also more frontal than Fz, although statistically the interaction effect did not reach significance (see Supplementary Figure S1). This inverse-U shape in the Manual theta power is difficult to interpret but could possibly point to some difference in the ability to regain vigilance after some time in manual driving, as the task is more engaging than PAD. Some studies have suggested that monotonous manual driving is safe up to a limit of 80 min (Ting et al., 2008). However, this is speculative and requires further investigation.

Frontal beta power also increased over time during driving, equivalently in both cap and trEEGrid data. Frontal beta has been shown to increase over time during cognitive tasks (Pershin et al., 2023; Tran et al., 2020), and driving (Craig et al., 2012). As increased beta power has been associated with increased mental activity or arousal (Andreassi, 2010), increased beta power over time has been interpreted as an increase in mental effort to counteract increasing fatigue levels caused by cognitive underload (Craig et al., 2012).

Significant differences from T1 were shown as early as T2 in the cap alpha and theta bands, as well as T3 for beta. In the grid significant differences relative to T1 appeared as of T3 for alpha and beta band activity. This demonstrates the potential of EEG data to detect vigilance decrements earlier or equivalently to PERCLOS (at T3), a frequently used measure of driver drowsiness (see Abe, 2023). However, as PERCLOS is an estimate of drowsiness and not necessarily vigilance, it's possible that spectral EEG is a more wholistic measure of decrements to driver vigilance and drowsiness than PERCLOS. Further, camera-based data such as PERCLOS depend on visual information and can be challenged by lighting conditions, as it relies on visibility to obtain information from the driver's eye. Both optimal illumination as well as good visibility of the eyes can not always be guaranteed. Additionally, spectral differences relative to the beginning were present at T3 or T4 for all measured EEG frequency bands in both cap and grid data, showing that changes in vigilance could be detected earlier in EEG data than in the RMSE lane deviation data (i.e., driving performance), which started to differ significantly from T1 only at T5 and T6, which corresponds to at least 10 min later or about 13 km when driving at 80 km/h.

Importantly, the results of the correlational analyses between the PSD values and lane deviation (Table 1) reveal the strong relationship between the neuromarkers analyzed in this study and actual behavioral performance during the simulated driving task. This association provides additional support for the relevance of EEG data as useful und informative methodology to obtain information on vigilance while performing an everyday task.

### The trEEGrid for EEG recording

This was the first study to use this version of the trEEGrid sensor system to investigate cognitive changes such as vigilance in a simulated real-world task such as driving. The EEG data recorded with the trEEGrid support the original hypotheses stated in the introduction: 1. PAD mode showed lower levels of vigilance compared to manual driving, 2. levels of vigilance decreased with time-on-task, 3. EEG changes linked to vigilance were detected earlier than behavioral driving parameters and about the same time as PERCLOS data, and 4. The effects detected with the 10–20 EEG cap are comparable to the effects recorded with the mobile trEEGrid. The last point highlights the value of the trEEGrid as it demonstrates that in certain use-cases and depending on the EEG parameters of interest, a mobile sensor system such as the trEEGrid is capable of providing high quality data without the burden of having to place and wear a full EEG cap. The comparability between grid solutions and 10–20 electrode locations has also been found in previous studies looking at different EEG parameters when comparing 10–20 sytem EEG channels with cEEGrid (Craig et al., 2012), and trEEGrid (Da Silva Souto et al., 2022). These results taken together underline the potential of the mobile EEG grid for research as well as applications in real-world settings where comfortable, unobtrusive, and easy to use EEG sensor systems are highly desirable. As shown here, this is relevant for driving, as well as in professional contexts, but solutions like the trEEGrid open possibilities to better understand and monitor mental processes in safety-critical work environments thereby helping to improve working conditions and safety aspects. Since behavior in real-world contexts is complex, an EEG based monitoring system would also benefit from additional parameters such as PERCLOS or performance markers to capture safety critical changes in mental states. Particularly in use-cases where safety is very critical a certain level of redundancy is necessary to make a system more failsafe.

### Limitations

This study used a driving simulator instead of real-car driving. There is some evidence for the validity of driving simulators in relation to real-world driving behavior (Risto and Martens, 2014; Shechtman et al., 2009), and even that effects of fatigue can be stronger in simulated driving (Philip et al., 2005). However, it is possible that the driving task was not as salient as it would be in a real car. Given the mobility of the here introduced EEG sensor system (trEEGrid), conducting EEG studies while driving in real scenarios can be easily realized.

Additionally, similar to many studies, our study sample may not have been large or diverse enough with respect to age to represent the driving population. Future studies should include larger sample sizes and participants in more diverse age groups, in order to better generalize changes in vigilance during driving to all drivers. Additionally, future studies could go beyond the average vigilance measures across all participants in order to assess the consistency and applicability of these effects at the individual level, as well as generalizability in real world scenarios.

There is evidence that eye blinks can influence EEG activity in the delta and theta band (Hagemann and Naumann, 2001) particularly in frontal electrodes. Even though ASR has been shown to reduce the impact ocular artifacts (Blum et al., 2019; Chang et al., 2018), it cannot be completely ruled out that remaining ocular activity might have contributed to the recorded theta activity. Future studies should investigate this further and control for contamination by eye-blinks. However, the effects observed for the beta and alpha band are likely not impacted by ocular activity (Hagemann and Naumann, 2001).

While this experiment was designed such that it taxes vigilance and the results reported are in line with effects observed in context of vigilance and fatigue, it cannot be ruled out that training effects could also play a role. However, the task itself is not too complex and all participants were given a practice session in the beginning to familiarize themselves with the task. Furthermore, by counterbalancing the task sequence we attempted to mitigate possible time-on-task training effects.

## Conclusions

The results indicate that changes in vigilance can be detected in brain activity earlier and possibly more accurately than in behavioral or other physiological measures. Consistent with the literature, spectral EEG data showed differences between the two driving conditions which related to decreased engagement and vigilance during PAD, as well as time-on-task effects which indicate vigilance and fatigue changes over time in both conditions. The trEEGrid recorded high quality EEG data and showed the same effect patterns compared to the 10–20 full-cap EEG-system. Even though effect sizes were slightly smaller for alpha and theta in the trEEGrid compared to the cap, both EEG sensor systems revealed the same significant differences between the two driving conditions as well as significant changes over time in the alpha, beta, and theta EEG frequency bands. This highlights the potential for smaller and more convenient mobile EEG solutions as vigilance monitoring technologies in the future, thereby enhancing driver safety and the safety of individuals in other safety-critical environments.

## Data Availability

The datasets presented in this article are not readily available because sharing of raw data was not included in the ethics statement. Requests to access the datasets should be directed to Axel Heinrich Winneke, axel.winneke@idmt.fraunhofer.de.
