# Supplementary material for: Mind the road: attention related neuromarkers during automated and manual simulated driving captured with a new mobile EEG sensor system
Source: Front Neuroergon. 2025 Mar 12;6:1542379. doi: 10.3389/fnrgo.2025.1542379 (PMC11937089; doi:10.3389/fnrgo.2025.1542379)
Supplement: Supplementary file 1 [file Data_Sheet_1.pdf]

Supplementary materials

S1: PSD Frontal Theta Cap Electrodes Fp1 and Fp2

Figure S1 (left) depicts theta power (in (V^2)/Hz dB) in the FP1 channel of the cap. Evident from the plot is higher theta power in the PAD than Manual condition, with the power increasing with time in the PAD condition, and an inverted 'U' shape in the Manual condition similar to the results of the FPz_M2 channel of the trEEGrid (see Figure 8). For theta at FP1 we found a significant main effect of Mode ($F(1,26) = 11.44$; $p = 0.003$; $\eta_p^2 = 0.300$), and Time ($F(2.24,58.36) = 9.903$; $p < 0.001$; $\eta_p^2 = 0.276$), but no significant interaction effect ($F(2.29,59.41) = 3.87$; $p = 0.116$; $\eta_p^2 = 0.077$).

Figure S1 (right) depicts theta power (in (V^2)/Hz dB) in the FP2 channel of the cap. Evident from the plot is higher theta power in the PAD than Manual condition, with the power increasing with time in the PAD condition, and an inverted 'U' shape in the Manual condition similar to the results of the FP1 channel and FPz_M2 channel of the trEEGrid (see Figure 8). For theta at FP2 we found a significant main effect of Mode ($F(1,26) = 7.46$; $p = 0.011$; $\eta_p^2 = 0.223$), and Time ($F(2.19,57.01) = 8.076$; $p < 0.001$; $\eta_p^2 = 0.237$), but no significant interaction effect ($F(2.46,63.89) = 2,14$; $p = 0.115$; $\eta_p^2 = 0.076$).

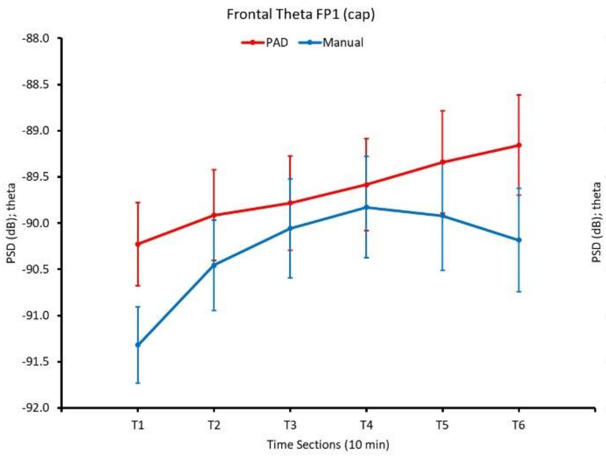

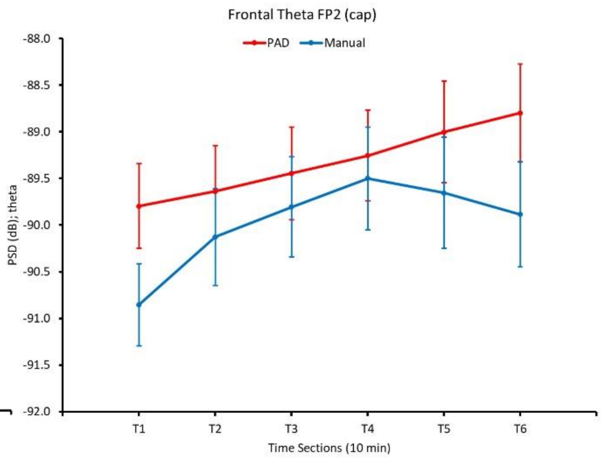

Figure S1. PSD plots for mean theta power (in (V^2)/Hz dB) for each of the six time sections with error bars indicating standard error in the measured electrode sites Fp1 (left) and Fp2 (right) of the EEG cap.

Measure	Comparison conditions (mean)	M_{diff}	*t-value*	df	*p-value*
**NASA TLX aggregated**	PAD(6.98) vs. Manual (8.16)	1.18	-2.69	27	0.012
**SSS**	Before (2.61) vs. After PAD (4.71)	2.11	5.73*	54	< 0.001
	Before vs. After Manual (4.29)	1.68	4.58*	54	< 0.001
	PAD vs. Manual	0.43	1.15*	54	0.26
**SSS-Q Engagement**	Baseline (3.61) vs. PAD (3.03)	0.58	4.08*	54	< 0.001
	Baseline vs. Manual (3.23)	0.38	2.35*	54	0.045
	PAD vs. Manual	0.2	1.73*	54	0.09
**SSS-Q Distress**	Baseline (1.14) vs. PAD (1.64)	0.5	4.18*	54	< 0.001
	Baseline vs. Manual (1.7)	0.56	4.25*	54	< 0.001
	PAD vs. Manual	0.06	0.07*	54	0.94
**RMSE Lane position**	Manual T1 (M = 0.53 m) vs. T5 (0.60 m)	0.076 m	-3.83	26	0.003
	Manual T2 (0.54 m) vs. T5	0.063 m	-3.18	26	0.022
	Manual T1 vs. T6 (0.62 m)	0.095 m	-4.75	26	<0.001
	Manual T2 vs. T6	0.082 m	-4.1	26	0.001
	Manual T3 vs. T6	0.059 m	-2.98	26	0.038
	Manual T4 vs. T6	0.057 m	-2.86	26	0.05
**PERCLOS**	PAD T1 (0.05) vs. T3 (0.088)	0.038	4.33*	125	< 0.001
	PAD T1 vs. T4 (0.081)	0.031	3.38*	125	0.012
	PAD T1 vs. T5 (0.077)	0.028	3.45*	125	0.01
	PAD T1 vs. T6 (0.11)	0.064	4.48*	125	< 0.001
	Manual T1 (0.019) vs. T3 (0.029)	0.01	3.67*	125	0.004
	Manual T1 vs. T4 (0.031)	0.011	3.67*	125	0.004
	Manual T1 vs. T5 (0.036)	0.017	5.21*	125	< 0.001
	Manual T1 vs. T6 (0.034)	0.015	6.09*	125	< 0.001
	Manual T2 (0.026) vs. T5	0.011	3.6*	125	0.005
	Manual T2 vs. T6	0.009	4.48*	125	< 0.001
	T1: PAD vs. Manual	0.031	3.87	25	< 0.001
	T2: PAD vs. Manual	0.043	3.89	25	< 0.001
	T3: PAD vs. Manual	0.059	3.73	25	< 0.001
	T4: PAD vs. Manual	0.05	3.58	25	< 0.001
	T5: PAD vs. Manual	0.041	3.89	25	< 0.001
	T6: PAD vs. Manual	0.08	3.56	25	< 0.001
**HRV**	Manual T1 (162.55) vs. T5 (201.93)	39.38	3.31*	130	0.018
	Manual T1 vs. T6 (192.86)	30.31	3.03*	130	0.042
**PVT**	Baseline (307.12 ms) vs. Session 1 (323.2 ms)	16.07 ms	-3.41	25	0.003
	Baseline vs. Session 2 (323.57 ms)	16.44 ms	-3.49	25	0.003
	Session 1 vs. Session 2	0.37 ms	-0.078	25	0.94
**Parietal Alpha: Grid**	PAD (-97.01 dB) vs. Manual (-98.25 dB) (all times)	1.24 dB	4.39	26	< 0.001
	PAD and Manual T1(-98.29 dB) vs. T3 (-97.53 dB)	0.76 dB	-4.15	26	< 0.001
	PAD and Manual T1 vs. T4 (-97.42 dB)	0.87 dB	-4.76	26	< 0.001
	PAD and Manual T1 vs. T5 (-97.39 dB)	0.91 dB	-4.95	26	< 0.001
	PAD and Manual T1 vs. T6 (-97.27 dB)	1.02 dB	-5.57	26	< 0.001
	PAD and Manual T2 vs. T6	0.6 dB	-3.27	26	0.015
**Parietal Alpha: Cap**	PAD (-97.01 dB) vs. Manual (-98.25 dB) (all times)	1.91 dB	8.14	26	< 0.001

	PAD and Manual T1 (-93.98 dB) vs. T2 (-93.3 dB)	0.68 dB	-4.86	26	< 0.001
	PAD and Manual T1 vs. T3 (-92.93 dB)	1.05 dB	-7.5	26	< 0.001
	PAD and Manual T1 vs. T4 (-92.86 dB)	1.12	-7.96	26	< 0.001
	PAD and Manual T1 vs. T5 (-92.80 dB)	1.18 dB	-8.39	26	< 0.001
	PAD and Manual T1 vs. T6 (-92.65)	1.33 dB	-9.47	26	< 0.001
	PAD and Manual T2 vs. T4	0.43 dB	-3.09	26	0.019
	PAD and Manual T2 vs. T5	0.5 dB	-3.53	26	0.005
	PAD and Manual T2 vs. T6	0.65 dB	-4.60	26	< 0.001
**Frontal Beta: Grid**	PAD (-96.25 dB) vs. Manual (-97.43 dB) (all times)	1.18 dB	3.65	20	0.002
	PAD and Manual T1 (-97.47 dB) vs. T3 (-96.63 dB)	0.84 dB	-3.95	20	0.002
	PAD and Manual T1 vs. T4 (-96.59 dB)	-96.59 dB	-4.12	20	0.001
	PAD and Manual T1 vs. T5 (-96.67 dB)	0.80 dB	-3.77	20	0.004
	PAD and Manual T1 vs. T6 (-96.74)	0.73 dB	-3.42	20	0.011
**Frontal Beta: Cap**	PAD (-97.3 dB) vs. Manual (-98.0 dB) (all times)	0.69 dB	3.17	26	0.004
	PAD and Manual T1 (-98.07 dB) vs. T3 (-97.46 dB)	0.61 dB	-5.46	26	< 0.001
	PAD and Manual T1 vs. T4 (-97.58 dB)	0.5 dB	-4.41	26	< 0.001
	PAD and Manual T1 vs. T5 (-97.59 dB)	0.48 dB	-4.26	26	< 0.001
	PAD and Manual T1 vs. T6 (-97.42 dB)	0.65 dB	-5.77	26	< 0.001
	PAD and Manual T2 vs. T6	0.34 dB	-3.04	26	0.032
**Frontal Theta: Grid**	PAD (-88.84 dB) vs. Manual (-89.99 dB) (all times)	1.14 dB	2.94	20	0.008
	PAD and Manual T1 (-90.08 dB) vs. T4 (-89.07 dB)	1.01 dB	-3.94	20	0.002
	PAD and Manual T1 vs. T5	0.90 dB	-3.49	20	0.01
	PAD and Manual T1 vs. T6	0.79 dB	-3.09	20	0.034
	Manual T1 (-90.80 dB) vs. T4 (-89.48 dB)	1.32 dB	-3.88	20	0.009
	T6 PAD (-88.23 dB) vs. Manual (-90.33 dB)	2.09 dB	4.34	20	0.005
**Frontal Theta: Cap**	PAD (-89.48 dB dB) vs. Manual (-90.03 dB) (all times)	0.55	2.82	26	0.009
	PAD and Manual T1 (-90.46 dB) vs. T2 (-89.96 dB)	0.50 dB	-4.41	26	< 0.001
	PAD and Manual T1 vs. T3 (-89.63 dB)	0.83 dB	-7.24	26	< 0.001
	PAD and Manual T1 vs. T4 (-89.59 dB)	0.87 dB	-7.63	26	< 0.001
	PAD and Manual T1 vs. T5 (-89.52 dB)	0.44	-8.22	26	< 0.001
	PAD and Manual T1 vs. T6 (-89.37)	1.09 dB	-9.53	26	< 0.001
	PAD and Manual T2 vs. T3	0.32 dB	-2.84	26	0.037
	PAD and Manual T2 vs. T4	0.37 dB	-3.22	26	0.013
	PAD and Manual T2 vs. T5	0.44 dB	-3.82	26	0.002
	PAD and Manual T2 vs. T6	0.59 dB	-5.12	26	< 0.001

Table S1. Test values for all post-hoc tests. df = degrees of freedom. * indicates a Conover post hoc comparisons test. Mean values are indicated in parentheses for the first instance of each condition. P-values are Bonferroni-Holm corrected (Abdi, 2010; Holm, 1979) and significant at < 0.05.
